# Remodeling and Tenacity of Inhibitory Synapses: Relationships with Network Activity and Neighboring Excitatory Synapses

**DOI:** 10.1371/journal.pcbi.1004632

**Published:** 2015-11-24

**Authors:** Anna Rubinski, Noam E. Ziv

**Affiliations:** Rappaport Faculty of Medicine, Network Biology Research Laboratories, Technion, Haifa, Israel; The Krasnow Institute for Advanced Studies, UNITED STATES

## Abstract

Glutamatergic synapse size remodeling is governed not only by specific activity forms but also by apparently stochastic processes with well-defined statistics. These spontaneous remodeling processes can give rise to skewed and stable synaptic size distributions, underlie scaling of these distributions and drive changes in glutamatergic synapse size “configurations”. Where inhibitory synapses are concerned, however, little is known on spontaneous remodeling dynamics, their statistics, their activity dependence or their long-term consequences. Here we followed individual inhibitory synapses for days, and analyzed their size remodeling dynamics within the statistical framework previously developed for glutamatergic synapses. Similar to glutamatergic synapses, size distributions of inhibitory synapses were skewed and stable; at the same time, however, sizes of individual synapses changed considerably, leading to gradual changes in synaptic size configurations. The suppression of network activity only transiently affected spontaneous remodeling dynamics, did not affect synaptic size configuration change rates and was not followed by the scaling of inhibitory synapse size distributions. Comparisons with glutamatergic synapses within the same dendrites revealed a degree of coupling between nearby inhibitory and excitatory synapse remodeling, but also revealed that inhibitory synapse size configurations changed at considerably slower rates than those of their glutamatergic neighbors. These findings point to quantitative differences in spontaneous remodeling dynamics of inhibitory and excitatory synapses but also reveal deep qualitative similarities in the processes that control their sizes and govern their remodeling dynamics.

## Author Summary

Synaptic plasticity is widely believed to constitute a fundamental mechanism for altering network function. An (implicit) extension of this belief is an assumption that spontaneous changes in synaptic function should not occur to any significant degree. Where excitatory synapses are concerned, recent studies have questioned the validity of this assumption. Where inhibitory synapses are concerned, however, much less is known. Here we followed the spontaneous remodeling dynamics of inhibitory synapses for days, and analyzed these dynamics within a statistical framework previously developed for glutamatergic synapses. Like their excitatory counterparts, sizes of individual synapses fluctuated considerably. Similarly, these spontaneous fluctuations were governed by a well-defined statistical process which assures that synaptic size distributions remain constant. Contrary to the aforementioned assumption, these spontaneous fluctuations drove changes in synaptic size configurations; interestingly, however, change rates were slower for inhibitory synapses. Unlike excitatory synapses, suppressing network activity barely affected inhibitory synapse remodeling dynamics, synaptic configuration change rates or synaptic size distributions. Our findings thus point to quantitative differences in spontaneous remodeling dynamics of inhibitory and excitatory synapses, but also indicate that the processes that control their sizes and govern their remodeling dynamics are fundamentally similar.

## Introduction

Activity-driven changes in synaptic properties are widely believed to constitute a fundamental mechanism for altering network function. This belief also (implicitly) implies that synapses, when *not* driven to change their properties by physiologically relevant stimuli, should retain these properties over time. Otherwise, physiologically relevant modifications would be gradually lost, or drowned in a sea of spurious changes.

The capacity of individual synapses to maintain their properties over behaviorally relevant time scales is by no means obvious: Imaging studies, carried over the last decade, have led to the realization that synapses are not structures in a strict sense but are better thought of as complex assemblies of dynamic components (receptors, scaffolding molecules, synaptic vesicles and organelles) which move in, out and between synaptic junctions on time-scales of seconds to hours [1,2]. Conceivably, these dynamics might challenge the capacity of synapses to maintain their individual properties over long time-scales (a capacity we refer to as *synaptic tenacity*). Indeed, when molecular contents of individual synapses are followed over many hours and days, these exhibit very considerable fluctuations, even in the absence of specific activity patterns, or, for that matter, any activity at all [3–15] (reviewed in [2]). Collectively these studies indicate that synapses exhibit significant spontaneous remodeling in addition to changes directed by specific activity patterns.

So far, synaptic tenacity has been studied mainly in the context of excitatory glutamatergic synapses. Where such synapses are concerned, these studies have pointed to several general principles: First, they suggest that whereas individual synapses exhibit significant spontaneous remodeling over long time scales, distributions of synaptic sizes are skewed and remarkably stable [5,7,14]. Second, it has been shown that spontaneous remodeling dynamics are altered by pharmacological manipulations of network activity, and that such changes can drive the scaling of synaptic size distributions [5,14]. Third, it has been shown that the spontaneous remodeling of individual synapses drives gradual changes in synaptic size configurations, such that over time scales of several days, relations with original synapse sizes are gradually lost [5,14] (a phenomenon we refer to here as a deterioration of synaptic size configurations). Finally, it has been shown that synaptic size dynamics and their effects on synaptic size distributions, scaling and synaptic configurations are described exceptionally well by a statistical process known in probability theory as the Kesten process [14].

At present, it remains unknown if these principles apply to inhibitory synapses as well. Like their excitatory counterparts, inhibitory synapses are dynamic assemblies, in which receptors—both glycine and γ-amino butyric acid (GABA) receptors—continuously diffuse in and out of synaptic sites (e.g. [16–19]; reviewed in [20,21]). These receptors are temporarily retained at synaptic sites through interactions with scaffolding molecules, mainly gephyrin (e.g. [16,22–25]) which, in turn, also exhibit substantial dynamics (e.g. [21,23,26,28–29]; reviewed in [1,20,21]). It thus might be asked: How tenacious are individual inhibitory synapses? How stable are inhibitory synapse size distributions? Are these rightward skewed as well? How are individual and population measures of synaptic tenacity affected by network activity levels? Is the suppression of network activity also associated with the scaling of inhibitory synapse size distributions? Do configurations of inhibitory neurons deteriorate over time? Are inhibitory synapse size dynamics also well-described by a Kesten process? And finally, how do spontaneous changes in inhibitory synapse sizes compare to spontaneous changes in excitatory synapse sizes? Do their size configurations deteriorate at similar rates?

To address these questions we used long-term imaging, multielectrode array recordings and fluorescently tagged gephyrin and PSD-95 to evaluate the spontaneous remodeling dynamics of inhibitory synapses and neighboring glutamatergic synapses, respectively. Our findings are described next.

## Results

Gephyrin is a highly conserved, widely expressed protein that is considered to be the core scaffolding protein at postsynaptic densities (PSDs) of inhibitory synapses [30,31]. In neurons, gephyrin plays key roles in the confinement of glycine and GABA-gated chloride channels to the postsynaptic membrane (e.g. 16, 22–24], and its synaptic content is considered to be a reliable indicator of inhibitory synapse size [31]. As gephyrin localizes very specifically to GABAergic (and glycinergic) synapses, fluorescently tagged variants of this molecule have been used *in vivo* and *in vitro* to visualize inhibitory synapses in living neurons. This approach has been used for studying synaptic targeting of gephyrin [33,34], inhibitory synapse formation [35] inhibitory synapse turnover following manipulations of network activity and sensory input [36–38], synaptic dynamism [26, 27], and activity-induced inhibitory synapse remodeling [28,29,38] (reviewed in [39]). In some of these studies [36–38], correlated light-electron microscopy of fluorescent gephyrin clusters established a tight correspondence between these fluorescent objects and inhibitory synapses identifiable at the ultrastructural level, validating the use of fluorescently tagged gephyrin as a highly reliable marker of inhibitory synapses.

Given its central role at inhibitory synapses, changes in gephyrin contents are likely to reflect changes in functional properties of the same synapses [21,39]. More conservatively, changes in gephyrin contents are very likely to reflect changes in the sizes of postsynaptic scaffolds at these synapses [32], and thus fluorescently tagged gephyrin can be used as a reporter of inhibitory synapse size. We thus created a fusion protein of a full length variant of gephyrin [23] and the bright cyan fluorescent protein variant mTurquoise2 [40], and used lentiviral vectors to sparsely express this fusion protein (mTurq2:Geph) in large networks of rat cortical neurons in primary culture. To follow these neurons over many hours and days, the neurons were grown on thin-glass multielectrode array (MEA) dishes, that in addition to allowing for long-term, high-resolution fluorescence imaging, allowed us to continuously record network activity in the same networks from the 59 extracellular electrodes embedded in these dishes [5,41].

**Fig 1:**
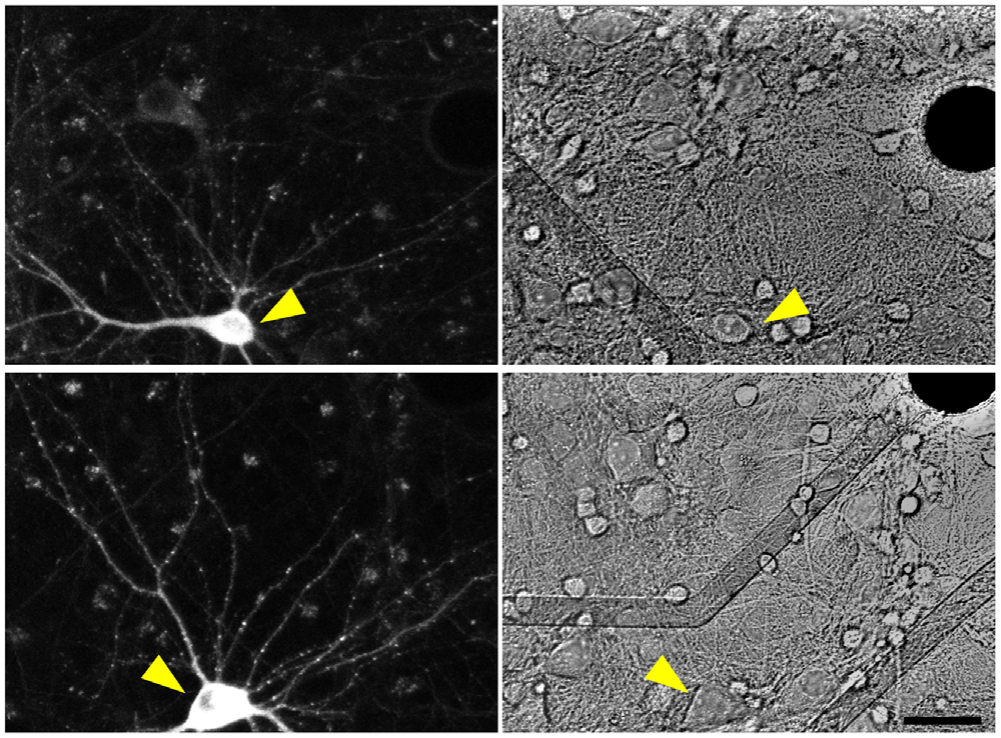
mTurq2:Geph expression in cortical neurons growing on MEA dishes. Fluorescence images of two neurons expressing mTurq2:Geph (left) and brightfield images of the same regions (right). Note the punctate distribution of mTurq2:Geph along dendritic shafts. The position of these neurons relative to nearby extracellular electrodes (opaque circles) and leads (transparent) is shown in the right-hand panels (yellow arrowheads). Bar, 30μm.

**Fig 2:**
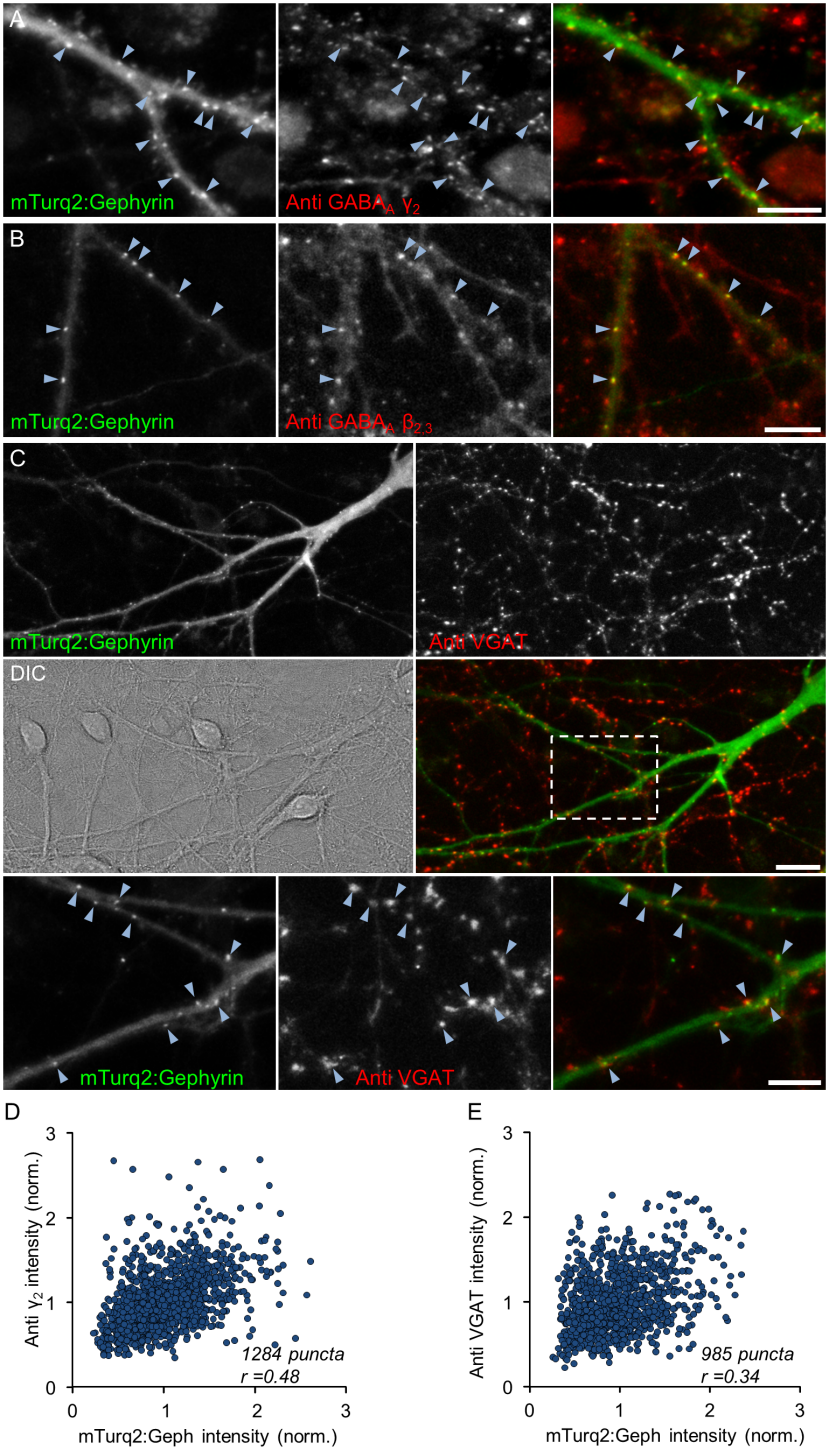
mTurq2:Geph colocalizes with GABA_A_ receptors and presynaptic boutons of GABAergic neurons. **A,B)** GABA_A_ receptors were labeled in live hippocampal neurons using antibodies against extracellular epitopes of the GABA_A_ receptor γ_2_ subunit (A) or GABA_A_ receptor β_2,3_ subunits (B). Left panels: mTurq2:Geph; Middle panels: antibody labeling; Right panels: combined images. Note the good colocalization of mTurq2:Geph puncta with clusters of labeled receptors (arrowheads point to some examples). Bars, 10μm. **C)** Functional presynaptic boutons of GABAergic neurons were labeled in live neurons using anti vesicular GABA transporters (VGAT) antibodies. Top panels—mTurq2:Geph (left) and anti-VGAT (right). Middle panels—differential interference contrast (DIC) image of the same field (left) and the combined image (right). Bottom panels—enlarged images of region enclosed in rectangle in right middle panel. Note the good colocalization of mTurq2:Geph puncta with clusters of labeled VGAT (arrowheads). Bars, 20μm (middle), 10μm (bottom). **D,E)** Correlation between mTurq2:Geph fluorescence and GABA_A_γ_2_ labeling (D; 3 Experiments, 18 cells, 1284 synapses) and VGAT labeling (E; 4 Experiments, 17 cells, 985 synapses).

As shown in Fig 1, mTurq2:Geph assumed a punctate appearance, in which fluorescent puncta were mainly distributed along weakly fluorescent dendritic shafts. As mentioned above, an excellent correspondence between such puncta and inhibitory synapses was previously reported. We confirmed these observations in two manners: We first performed labeling of live neurons using two different antibodies raised against extracellular epitopes of the most common subunits of postsynaptic GABA_A_ receptors, namely γ_2_ and β_2,3_ [30] and compared the resulting labeling pattern with those of mTurq2:Geph in the same neurons (see Materials and Methods for further details). Live labeling was performed at 14–18 DIV in sparse networks of cultured hippocampal neurons rather than cortical neurons because the lower cell and synapse density facilitated antibody access and colocalization analysis. As shown in Fig 2A and 2B an excellent degree of colocalization of mTurq2:Geph with clusters of labeled receptors was observed (89% of mTurq2:Geph puncta colocalized with GABA_A_ γ_2_, 3 experiments, 18 Cells, 1284 puncta; 91% of mTurq2:Geph puncta colocalized with β_2,3_ receptor clusters, one experiment, 4 Cells, 252 puncta; colocalization with images rotated 180°: 14% and 12%, respectively). On the other hand, when mTurq2:Gephyrin and antibody labeling fluorescence were compared on a synapse to synapse basis, the correlation, although positive, was quite imperfect (*r* = 0.48, Fig 2D).

We then examined the colocalization of mTurq2:Geph with functional presynaptic boutons of GABAergic neurons by visualizing the uptake of antibodies directed against the lumenal domain of the vesicular GABA transporter (VGAT) as previously described [42]. We found that 71% of mTurq2:Gephyrin clusters colocalized with VGAT puncta (Fig 2C; 4 Experiments, 17 cells, 985 puncta; colocalization with images rotated 180°: 20%). Although this colocalization was less than that observed for GABA_A_ receptors, it is important to keep in mind that presynaptic labeling in this assay depended on the spontaneous activity levels of GABAergic neurons whose boutons impinged on the mTurq2:Geph-expressing neurons, and it is unlikely that these were all equally active. Here too we noted that the correlation between VGAT uptake and mTurq2:Geph fluorescence at single synapses was positive but imperfect (*r* = 0.34; Fig 2E).

In summary, the good colocalization between mTurq2:Geph, GABA_A_ receptor clusters and VGAT-positive presynaptic boutons further supports prior observations that mTurq2:Geph puncta correspond to *bona-fide* GABAergic synapses. On the other hand, we did note that the quantitative correlation between the three, when examined on a synapse to synapse basis, was not perfect. This limited correlation might reflect imperfect antibody labeling (due to poor penetration into the synaptic cleft, for example), substantial GABA_A_ receptor composition heterogeneity [43], the heterogeneity of pre/post synaptic “stoichiometry” [12] or differences in spontaneous activity levels. Thus, while some uncertainty remains as to quantitative relationships between mTurq2:Geph fluorescence intensity at a particular synapse and functional measures of the same synapse, mTurq2:Geph can be used to measure the constancy of gephyrin contents at identified inhibitory synapses and by extension, estimate the constancy of their sizes.

### Inhibitory synapse size distributions are skewed, stable and do not scale following network activity suppression

**Fig 3:**
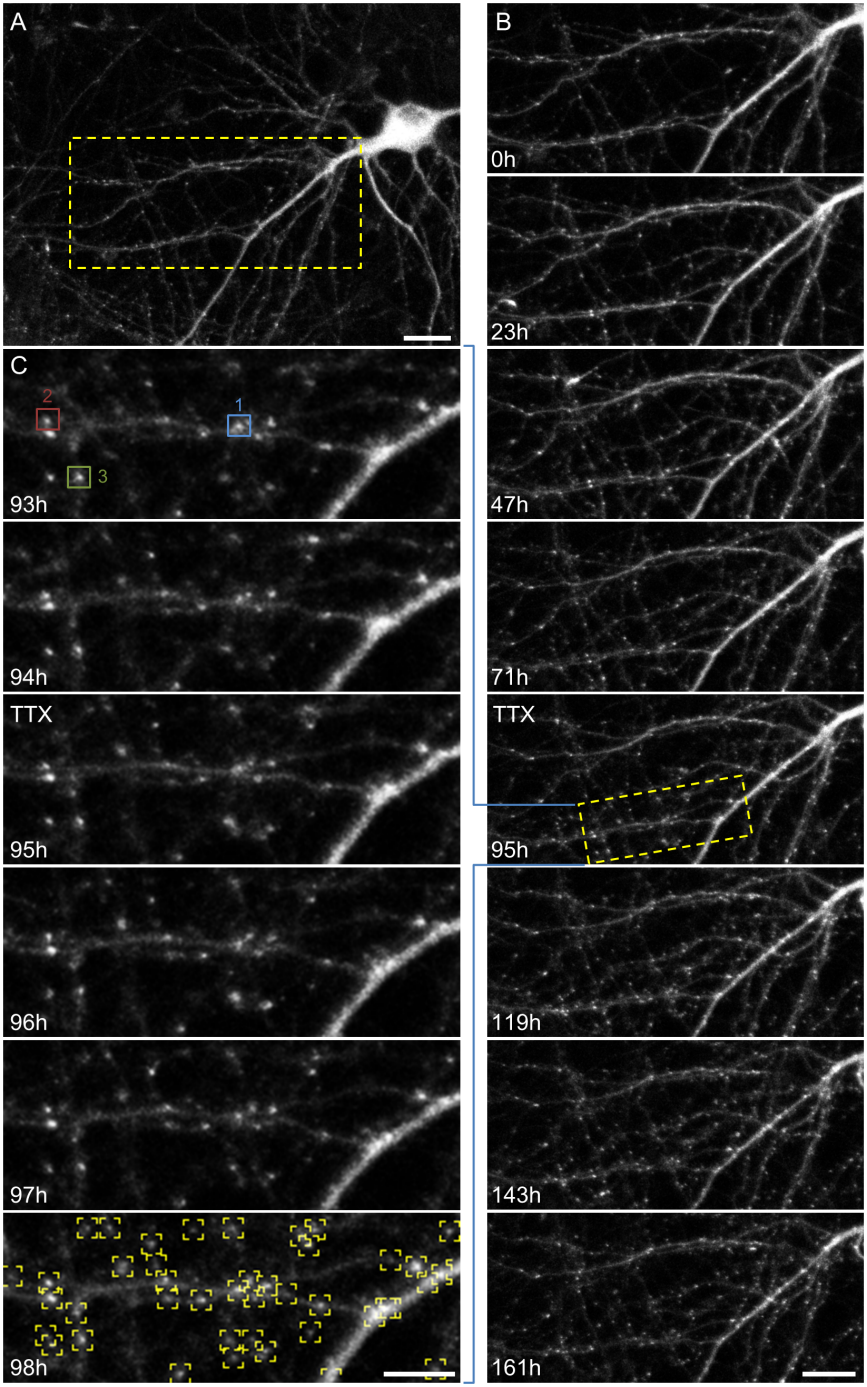
Long term imaging of mTurq2:Geph puncta. **A)** A rat cortical neuron expressing mTurq2:Geph within a network of cortical neurons growing on a thin-glass MEA dish (starting at 18 days *in vitro*). Bar, 20μm. **B)** Time-lapse imaging of the region enclosed in rectangle in (A). Images were collected at one hour intervals for 161 hours (~7 days). Only a subset of the data is shown here. Bar: 20μm. **C)** High temporal and spatial resolution images of region enclosed in rectangle in (B). Note the excellent ability to follow the same synapses over time. Fluorescence measurement data for the three synapses enclosed in colored rectangles is provided in Fig 5A. The bottom panel demonstrates the programmatic detection of mTurq2:Geph puncta, used thereafter for counting and fluorescence quantification. Bar, 10μm.

**Fig 4:**
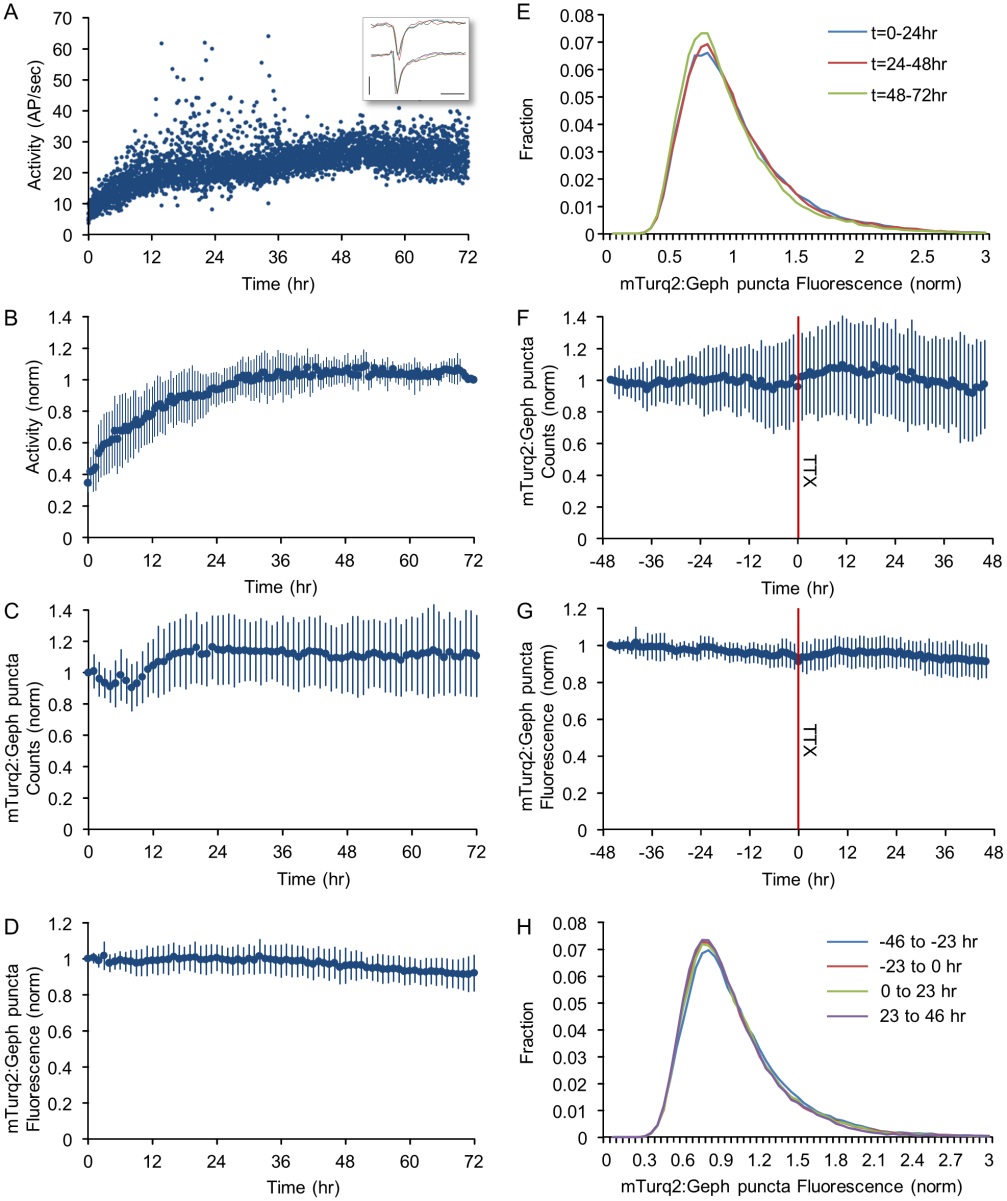
Population measures of inhibitory synapse sizes and numbers are stable and only modestly affected by the suppression of spontaneous activity. **A)** Spontaneous activity recorded for 72 hours in one network, starting with the mounting of the preparation on the combined MEA recording/imaging system. Every dot represents the total number of action potentials (AP) recorded from all electrodes during a one minute period, divided by 60. Action potentials were identified on-line as threshold crossing events (with the threshold set at -0.4 mV), in traces such as those shown in the inset (6 action potentials recorded from two electrodes). Bars, 1msec, 1mV. **B)** Changes in spontaneous activity levels—pooled data from 6 experiments. Data was normalized to activity levels measured at t = 72 hours. **C)** Changes in mTurq2:Geph puncta numbers pooled from 27 neurons in 4 experiments in which images were obtained at one hour intervals. Counts for each neuron were normalized to initial puncta counts at t = 0. **D)** Changes in mTurq2:Geph fluorescence intensities pooled for the same 27 neurons as in C). Fluorescence values were normalized to mean fluorescence at t = 0. **E)** Distributions of mTurq2:Geph puncta fluorescence intensities averaged over three consecutive, 24-hour windows for the same 27 neurons as in C,D). **F)** Changes in mTurq2:Geph puncta numbers following the abrupt suppression of network activity by exposing the networks to TTX at t = 0 (red line, see also S3 Fig). **G)** Changes in mean mTurq2:Geph puncta fluorescence intensities following the suppression of network activity at t = 0. **H)** Distributions of mTurq2:Geph puncta fluorescence intensities during 23-hour consecutive time windows before and after exposure to TTX (4 Experiments, 27 neurons, ~4,000 synapses). All values in B-D,F,G represent means ± standard deviations.

We [5,14] and others (e.g. [7]) have previously shown that glutamatergic synaptic size distributions are rightward skewed (heavy tailed), stable over days, and exhibit scaling upon a suppression of spontaneous network activity. Are inhibitory synapse size distributions similar in these regards? To address this question, cortical networks maintained in culture for 18–21 DIV, were mounted on a combined MEA recording/imaging system [5,14,41], and followed for several days, during which neurons expressing mTurq2:Geph were imaged periodically by automated time lapse microscopy. During these experiments the preparations were maintained at optimal conditions provided by covering the MEA dish with a custom built cap equipped with inlet/outlet ports, streaming a sterile mixture of 5% CO_2_ and 95% air into the dish, perfusing the preparation slowly with fresh feeding medium and heating the MEA dish and oil-immersion objective to 37°C. These conditions were essential for maintaining the long term vitality of these preparations, allowing us to carry out experiments lasting one week or more with no signs of deterioration or cell death (Fig 3). Stacks of images (at 8 focal planes) of neurons expressing mTurq2:Geph were collected automatically from 6–10 fields of view (or sites), with each site representing a portion of the dendritic arbor of a different neuron. Images were collected at one hour intervals for several days concomitantly with recordings of network activity (action potentials, Fig 4A, inset) from the 59 electrodes of the MEA dish. In agreement with many prior studies from multiple groups ([5,41] and references therein), activity in these preparations tended to occur as spontaneous, network-wide bursts (S1 Fig). Although activity levels were generally quite stable, the first 24–36 hours of each experiment were invariably associated with a substantial increase in spontaneous activity levels (reflecting both increasing numbers of active neurons and increased firing rates), as shown for one representative experiment in Fig 4A and for data pooled from 4 experiments in Fig 4B. This initial increase in activity almost certainly reflects the introduction of the slow perfusion [5] and thus reflects an initial transient related to the experimental conditions of these long term experiments.

To quantify the stability of population measures of inhibitory synapses under baseline conditions, fluorescent mTurq2:Geph puncta in the image series were detected programmatically (Fig 3C, bottom panel) counted and their fluorescence was quantified (see Materials and Methods for further details). Such counts and fluorescence levels for one experiment are shown in S2A and S2B Fig (6 neurons, ~260 synapses) and for data pooled from all experiments in Fig 4C and 4D (4 experiments, 27 neurons, ~4,000 synapses). This analysis revealed that puncta counts were generally stable apart from a small initial increase in mTurq2:Geph puncta numbers which became apparent after the first 12 hours (Fig 4C; see also [44–47]). Over these same periods, average fluorescence intensities of mTurq2:Geph puncta were generally stable apart from a slight decrease over time (Fig 4D). The distributions of mTurq2:Geph puncta fluorescence (averaged over three consecutive, 24 hour windows) revealed a majority of small (dim) puncta with a long tail of increasingly brighter (larger) puncta (Fig 4E). These rightward skewed distributions were remarkably stable and showed only a very modest change over time that was consistent with the slight decrease in average mTurq2:Geph puncta fluorescence (Fig 4D). Importantly, this modest decrease in mTurq2:Geph fluorescence was not due to photobleaching, as the same trend was observed in networks that were imaged only once every 24 hours (S2C Fig; 2 experiments, 16 neurons).

We then set out to examine whether blocking all spontaneous activity in these networks would affect population measures of inhibitory synapse size. Will such a manipulation (homeostatically?) downscale inhibitory synapse size distributions, or perhaps drive an upward scaling, consistent with increases in inhibitory synaptic sizes observed under some conditions [28]? (see also [44]) To address this question, we suppressed network activity by adding tetrodotoxin (TTX; 1 μM) to the MEA dish and perfusion media. We then compared recordings carried out for 46–48 hours before TTX application (starting at least 24 hours after mounting the preparations) with those collected for 46–48 additional hours in the presence of TTX.

Pooling data from a total of 4 experiments showed that activity levels during the periods that preceded TTX application were relatively stable, and that, as expected, TTX led to the abrupt transition from very high activity levels to complete and sustained silence (S3A Fig). The cessation of network activity was associated with slight trends, observable over the first 12 hours from TTX treatment, towards increases in mTurq2:Geph puncta numbers (Fig 4F), and average mTurq2:Geph fluorescence values (Fig 4G), but these were not statistically significant. In agreement with these observations, distributions of mTurq2:Geph puncta fluorescence were barely affected (Fig 4H). Of note, practically identical results were obtained in an additional experiment in which Bicuculline (6μM) was added together with TTX (8 neurons, ~210 synapses; S4 Fig).

The lack of changes in gephyrin contents does not necessarily imply that synaptic GABA receptor contents did not change [48]. To test whether activity blockade influences GABA_A_ receptor contents we compared the labeling against GABA_A_ subunit γ2 in live neurons (as described in Fig 2A) either exposed or unexposed to TTX for 24–48 hours. In these experiments (4 experiments, 55 fields of view, >7000 synapses) we observed a trend toward an increase in anti γ2 labeling following a 24 hour exposure period to TTX (~9%, on average), but this increase was not statistically significant (P> 0.19; paired two-tailed t test).

Thus, like their excitatory counterparts, distributions of inhibitory synapses sizes were skewed and very stable over hours and even days. Unlike these, however, abrupt suppressions of network activity were not associated with notable scaling of inhibitory synapse size distributions in one direction or another.

### Individual inhibitory synapse sizes exhibit significant activity-independent changes

**Fig 5:**
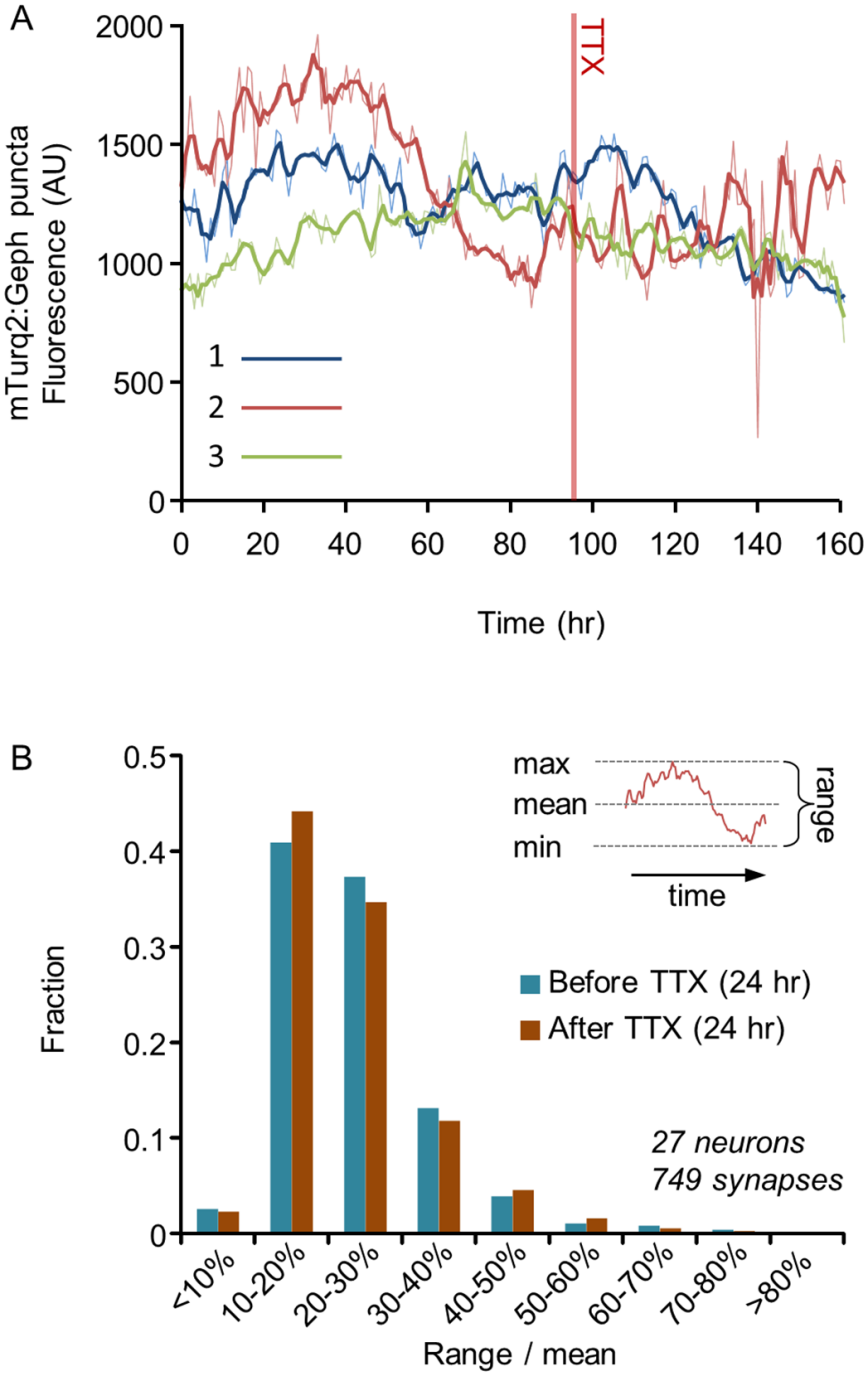
Spontaneous remodeling of individual inhibitory synapses. **A)** Changes over time in the fluorescence intensity of three mTurq2:Geph puncta (enclosed in color-coded rectangles in Fig 3C) measured over the course of 161 hours. Raw data is shown as thin lines whereas thick lines show the same data after smoothing with a 3-point low-pass filter. The filtered data was used in all subsequent analyses. **B**) Distributions of the range of changes exhibited by individual mTurq2:Geph over the course of 24 hours, before and after exposure to TTX (4 Experiments, 27 neurons, 749 synapses). The ranges of change of individual puncta were expressed as “range over mean” calculated as illustrated in inset, that is, by subtracting the minimal intensity from the maximal intensity and dividing this difference (the range) by the mean fluorescence intensity (eq 1 in main text).

As mentioned in the introduction, where glutamatergic synapses are concerned, the stability of synaptic size population coexists with significant fluctuations in the sizes of individual synapses within the same populations [5,7,14]. To examine if this holds true for inhibitory synapses as well, we tracked specific mTurq2:Geph puncta at one hour intervals and measured how their fluorescence changes over the time course of several days. For this analysis, only puncta present throughout the entire experiment were considered. This process is exemplified for 3 synapses in Figs 3C and 5A. To minimize effects of short term fluctuations and measurement noise, fluorescence intensities measured for each synapse were smoothed using a three-point (3 hour) low-pass-filter. As shown in Fig 5A, some puncta exhibited significant changes in their fluorescence over such periods, whereas the fluorescence of other puncta remained quite stable. Importantly, changes in puncta fluorescence continued to occur even when activity was blocked by TTX.

To quantify the extent of change exhibited by individual mTurq2:Geph puncta and compare these in spontaneously active and silenced networks, we calculated, for the filtered data of each tracked synapse, the normalized range of fluorescence change (“Range over Mean”; [8]) as illustrated in Fig 5B (inset)

$$\frac{Range}{Mean}=100*\frac{F_{max}-F_{min}}{\bar{F}}$$ (1)

where *F*_*max*_, *F*_*min*_ and $\bar{F}$ are the maximal, minimal and average fluorescence intensities measured for a given synapse over a given period, respectively. Average range over mean values measured over 24 hour periods were 24±10% (before TTX) and 24±13% (after TTX). Distributions of range over mean values before and after TTX are shown in Fig 5B (4 Experiments, 27 neurons, 749 tracked synapses). These distributions were rightward skewed, with about 20% of the puncta exhibiting changes of 30% or more over this period, and not different for active and silenced networks (P > 0.97; paired two tailed t-test).

These findings show that in common with what was observed for glutamatergic synapses, the stability of inhibitory synaptic size populations coexists with significant fluctuations in individual synapse sizes within the same populations. Moreover, the finding that the extent of such fluctuations is not different in highly active and completely silent networks indicates that these fluctuations are largely activity independent.

### Spontaneous changes in synaptic sizes lead to a gradual deterioration of inhibitory synapse “configurations”

**Fig 6:**
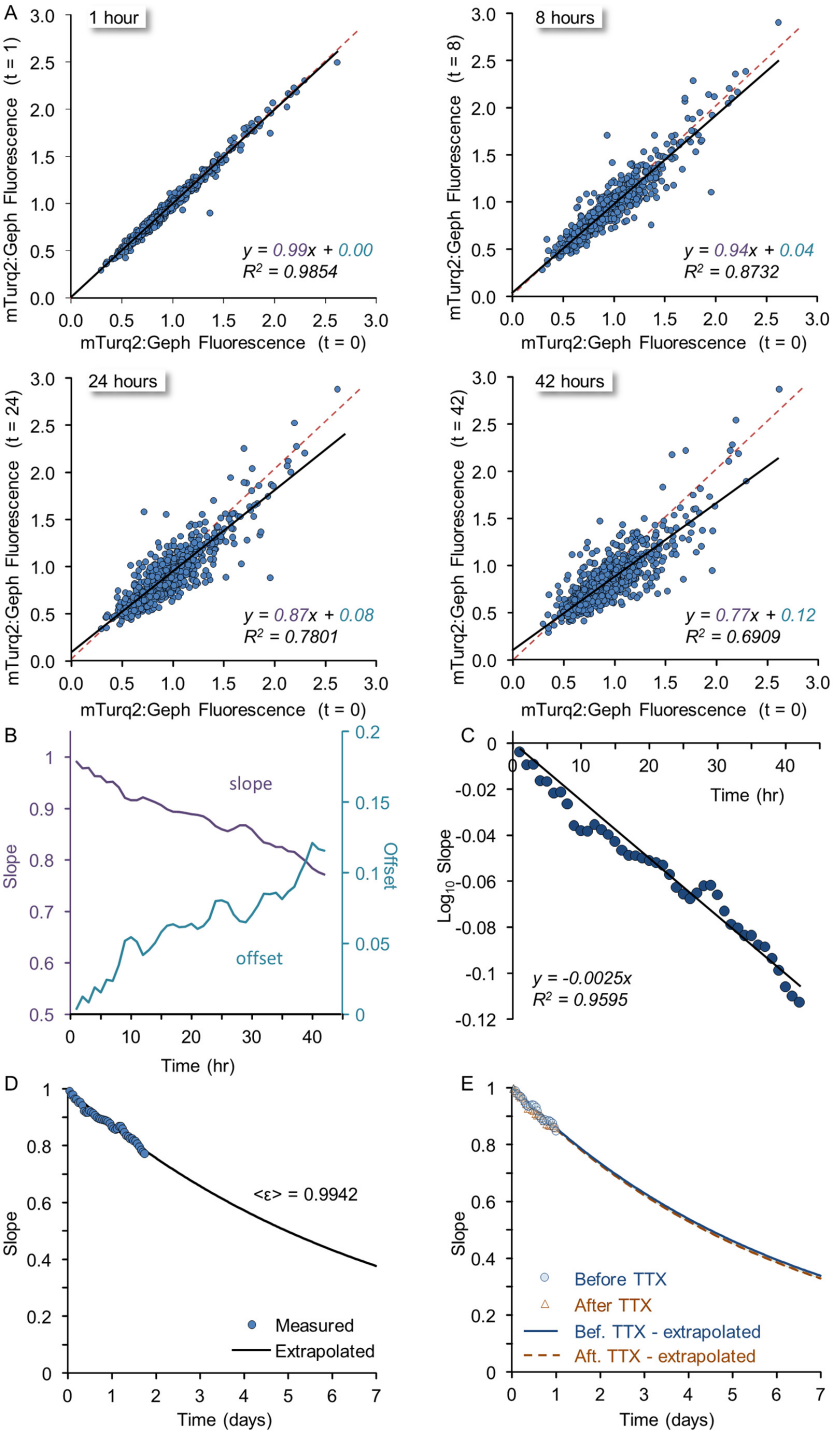
Spontaneous remodeling of individual inhibitory synapses analyzed in the framework of a Kesten process. **A)** The fluorescence of individual mTurq2:Geph puncta at increasing times (1,8,24 and 42 hours) as a function of their fluorescence at an arbitrary reference time point (t = 0). Linear regression lines are shown in black, whereas the unity line is shown as a dashed red line. Note that with time, the slope grows shallower, the offset increases and the goodness of fit (R^2^) decreases. 749 puncta from 27 neurons. **B)** The slopes and offsets of linear regressions such as those shown in (A) for all one-hour intervals from t = 1 to t = 42 hours). **C)** Obtaining the average value of ε (i.e. <ε>). According to eq 3 (main text) the slope in linear regression plots such as those shown in (A) should be 〈*ε*〉^*k*^ where k is the number of steps (hours, in this case); therefore log(*slope*) = log〈*ε*〉 ∙ *k*. Thus, when log(*slope*) is plotted as a function of *k* as shown here, log〈*ε*〉 can be estimated from a linear regression line in this plot (black), giving log〈*ε*〉 = -0.00253 and 〈*ε*〉 = 0.9942. **D)** Extrapolation of the slopes expected in plots such as those shown in (A) for longer durations. The expected slopes were calculated as (0.9942)^k^ where *k* is given in hours from t = 0. The empirical data shown in (B) and (C) is overlaid onto the calculated curve. **E)** Slopes and <ε> are similar in spontaneously active networks and in networks in which activity was blocked for 24 hours. Analysis similar to that outlined in B-D was performed for two time windows before (-25 to -1 hours) and after (22 to 46 hours) exposure to TTX. The derived values of <ε> were essentially indistinguishable.

What is the cumulative effect of fluctuations in synaptic sizes? One possibility is that the observed fluctuations have no cumulative effect, merely reflecting, for each synapse, fluctuations around some mean size, such that on average, synaptic sizes remain constant. Put differently, these fluctuations might have no lasting impact on configurations of inhibitory synapse sizes for particular neurons. To examine how such fluctuations might affect configurations of inhibitory synaptic sizes, we followed individual synapses in spontaneously active networks (same neurons as those used for obtaining the data in Fig 4) and plotted the mTurq2:Geph fluorescence of each synapse (749 synapses from 27 neurons in 4 experiments) at increasing times against its fluorescence at an initial time point. We selected this initial time point to be t = 30h to move away from the transient observed at the beginning of such experiments (Fig 4C). As shown in Fig 6A, over the course of 42 hours, the correlation between initial and subsequent mTurq2:Geph fluorescence gradually decreased (manifested as decreases in the coefficient of determination, or R^2^, serving as a goodness of fit measure). More importantly, however, the slopes of linear regression lines for these plots gradually decreased, whereas their offsets gradually increased (Fig 6B). These systematic and monotonic changes in regression line parameters are not consistent with random fluctuations around fixed points. In contrast, they are very much in line with a statistical framework we recently formulated for glutamatergic synapse remodeling [14] which is based on a statistical process known as the Kesten process [49–51].

The basic premise of the aforementioned framework is that synaptic remodeling dynamics are governed by both additive and multiplicative processes, both of which are inherently stochastic (but can be parametrically dependent on activity) and represent the aggregate of multiple microscopic molecular processes. According to this model, for a given synapse of size *x*, its new size after some discrete time period (i.e. at t+1) will be

$$x_{t+1}=\;\varepsilon_{t}x_{t}+\eta_{t}$$ (2)

where ε_t_ and η_t_ are not fixed values but *random variables* drawn independently at each time step from some distribution. In this process, which can give rise to rich and complex dynamics, if ε_t_ is, on average less than 1.0 (or more accurately, if 〈ln ε〉 < 0) and η_t_ is on average positive, size distributions will be both stable and rightward skewed as in Fig 4E and 4H, even though sizes of individual objects fluctuate continuously. Furthermore, it can be shown [14] that when the distributions are stable, the iteration of this process *k* times will give rise to objects whose size 〈*x*_*k*_〉 on average is such that

$$\langle x_{k}\rangle =\;{\langle \varepsilon \rangle }^{k}\;x_{t=0}+\left(1-{\langle \varepsilon \rangle }^{k}\right)$$ (3)

where 〈*ε*〉 is the average value of ε for the discrete time steps used, and x_t = 0_ is the initial size of the object. Note that in this solution, the slope 〈*ε*〉^*k*^ decreases as *k* increases (recall that 〈*ε*〉 < 1), whereas the offset (1−〈*ε*〉^*k*^) increases. Indeed, this is what we observed for the slopes and offsets of regression lines (Fig 6A and 6B) when the fluorescence values of mTurq2:Geph puncta *x*_*k*_ were plotted against their initial fluorescence (*x*_*t = 0*_) at increasingly greater time intervals (*k*). Moreover, plotting theses slopes as a function of the time interval (*k*) allowed us to estimate 〈*ε*〉 (= 0.9942) as illustrated in Fig 6C and then extrapolate the expected slopes for increasingly longer periods (Fig 6D). The estimate of 〈*ε*〉 and its extrapolation can then be used to estimate the expected rates of synaptic configuration deterioration; In this case, this extrapolation indicated that within a week, the slope would be reduced to 0.38 and within two weeks to 0.14 (not shown). The expected offset at that time would be ~0.9 (eq 3). It should be noted, however, that as mean mTurq2:Geph fluorescence slightly declined over these periods (Figs 4D and S2C), a small component of reduced slope values might be attributable to this slight decline. Correcting for this resulted in extrapolated slopes of 0.59 and 0.35 after one and two weeks, respectively.

Coefficient of determination (R^2^) values were more difficult to extrapolate as they depend on accurate estimates of the particular distributions of ε and η which are harder to obtain [14] but the empirical data suggested that R^2^ values decayed quite rapidly (from 0.99 after one hour to 0.69 after 42 hours; Fig 6A), indicating that after 1–2 weeks R^2^ values would be very small. Taken together with the changes in slopes and offsets described above, the data indicate that within 1–2 weeks, relationships with original synaptic configurations would be largely lost.

To examine if the suppression of network activity affected these decay rates, we performed a similar analysis for the same synapses after exposure to TTX. Specifically, we compared 24 hour time windows before (from -25 to -1 hours) and after (from 22 to 46 hours) adding TTX to the dishes and media. As shown in Fig 6E, the rate at which the slope decayed in these two time periods was practically identical.

**Fig 7:**
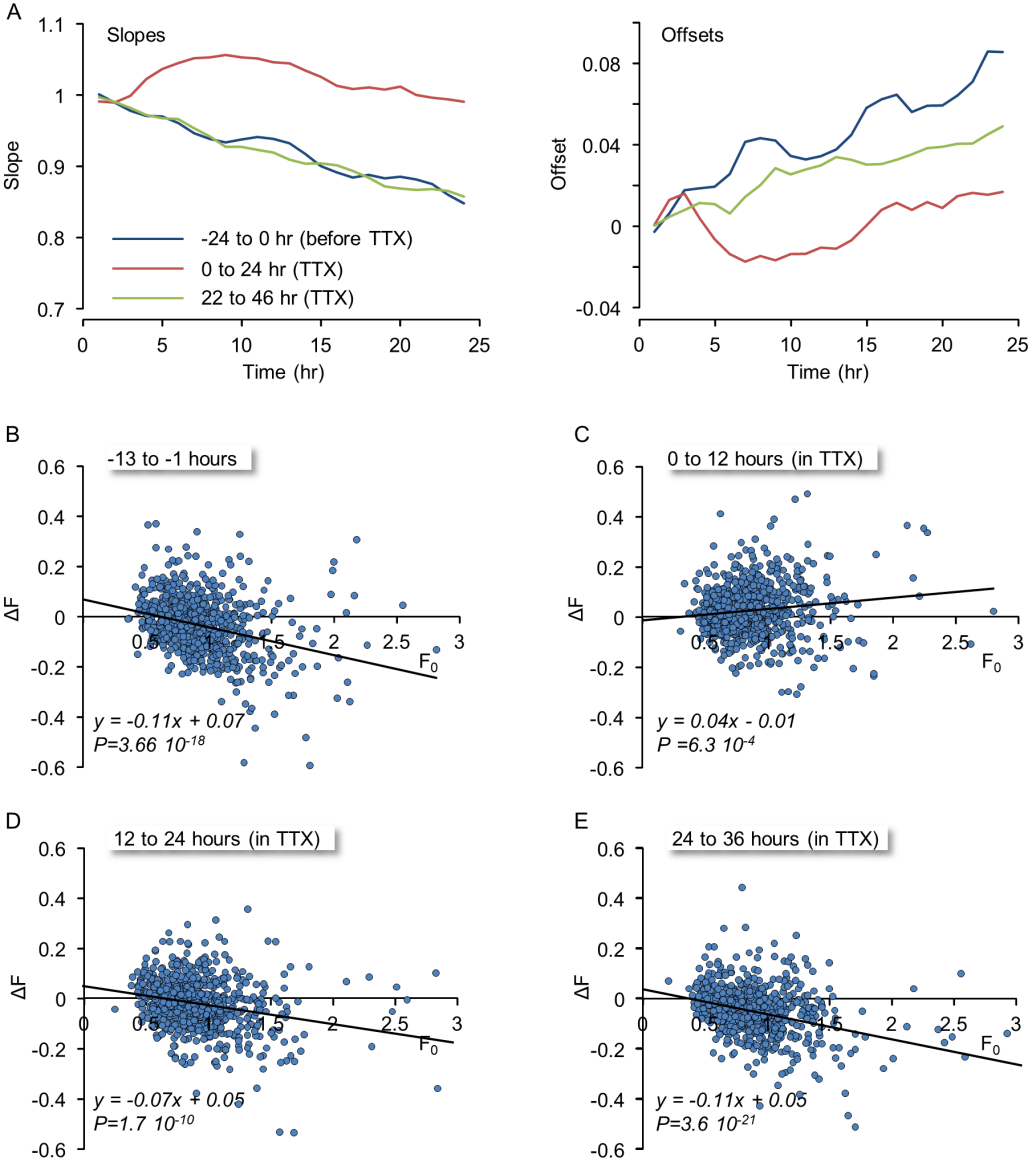
Suppression of spontaneous activity leads to transient changes in remodeling dynamics. **A)** The slopes and offsets of linear regressions similar to those shown in Fig 6A for three consecutive 24 hour periods beginning 24 hours before exposure to TTX, immediately after exposure to TTX and 22 hours after exposure to TTX. Note that immediately after exposure to TTX, the slopes increase and offsets decrease, but these effects are temporary and mostly gone after 12 hours. **B-E)** Changes in the fluorescence of individual mTurq2:Geph puncta as a function of their initial fluorescence during 12 hour windows before (B) and after (C-E) exposure to TTX. ΔF was calculated by subtracting the fluorescence at the beginning of each time window (F_0_) from the fluorescence at its end. Solid lines are linear regression fits. P denotes the statistical significance of the regression lines. Note the transient change in the trends of the regression line immediately after TTX addition (C) and the recovery in subsequent time windows (D,E).

Although the findings described so far would seem to indicate that blocking network activity had little effect on the spontaneous remodeling dynamics of inhibitory synapses, we did observe transient effects that mostly disappeared after <24 hours. The first manifestation of this transient was in the evolution of the slopes and offsets in plots such as those shown in Fig 6. Whereas the slopes and offsets before exposure to TTX and 24 hours after application were quite similar, transient increases and decreases in these slopes and offsets, respectively, were observed during the first 12 hours following TTX application (Figs 7A and S5). This transient was also apparent when *changes* in the mTurq2:Geph fluorescence of individual puncta were plotted as a function of their initial fluorescence. Specifically, changes in the fluorescence intensity of each synapse at the end of 12 hour time windows were calculated by subtracting the fluorescence at the beginning of each time window (F_0_) from the fluorescence at the end of the time window, and these changes (ΔF) were then plotted as a function of F_0_ (Fig 7B–7E). As shown here, significant changes in puncta fluorescence were observed for most puncta, regardless of their initial fluorescence. In addition, and in common with previous reports for glutamatergic synapses [5,41] and as expected for changes governed by the Kesten process [14], consistent relationships were observed between the direction of change and initial fluorescence, with the largest synapses showing a tendency to grow smaller, the smallest synapses a tendency to grow larger, and regression lines exhibiting negative slopes and a tendency to cross the abscissa near the mean fluorescence value. Interestingly, however, in first 12 hour window following TTX application, these tendencies were transiently reversed (Fig 7C), only to recover during the next time windows (Figs 7D, 7E and S5).

Collectively, these data suggest that spontaneous inhibitory synapse remodeling leads to a gradual deterioration of inhibitory synapse size configurations, to the point that in these preparations, these are lost, for the most part, after 1–2 weeks; Moreover, apart from transient effects, the data indicate that the deterioration of inhibitory synapse size configurations occurs in highly active and silent networks to a similar extent.

### Spontaneous remodeling dynamics of neighboring inhibitory and excitatory synapses

The findings described above indicate that in common with what was observed for glutamatergic synapses [5,14] configurations of inhibitory synapses deteriorate over time. Are configuration deterioration rates identical for these two major types of synapses? A comparison of estimates of 〈*ε*〉 (for one-hour time intervals) for glutamatergic synapses (0.9848; [14]) and inhibitory synapses (0.9942; Fig 7C) indicates that configurations of inhibitory synapses deteriorate at a slower pace than configurations of glutamatergic synapses. This comparison is based, however, on data collected in different sets of neurons at different times, questioning the validity of this comparison.

**Fig 8:**
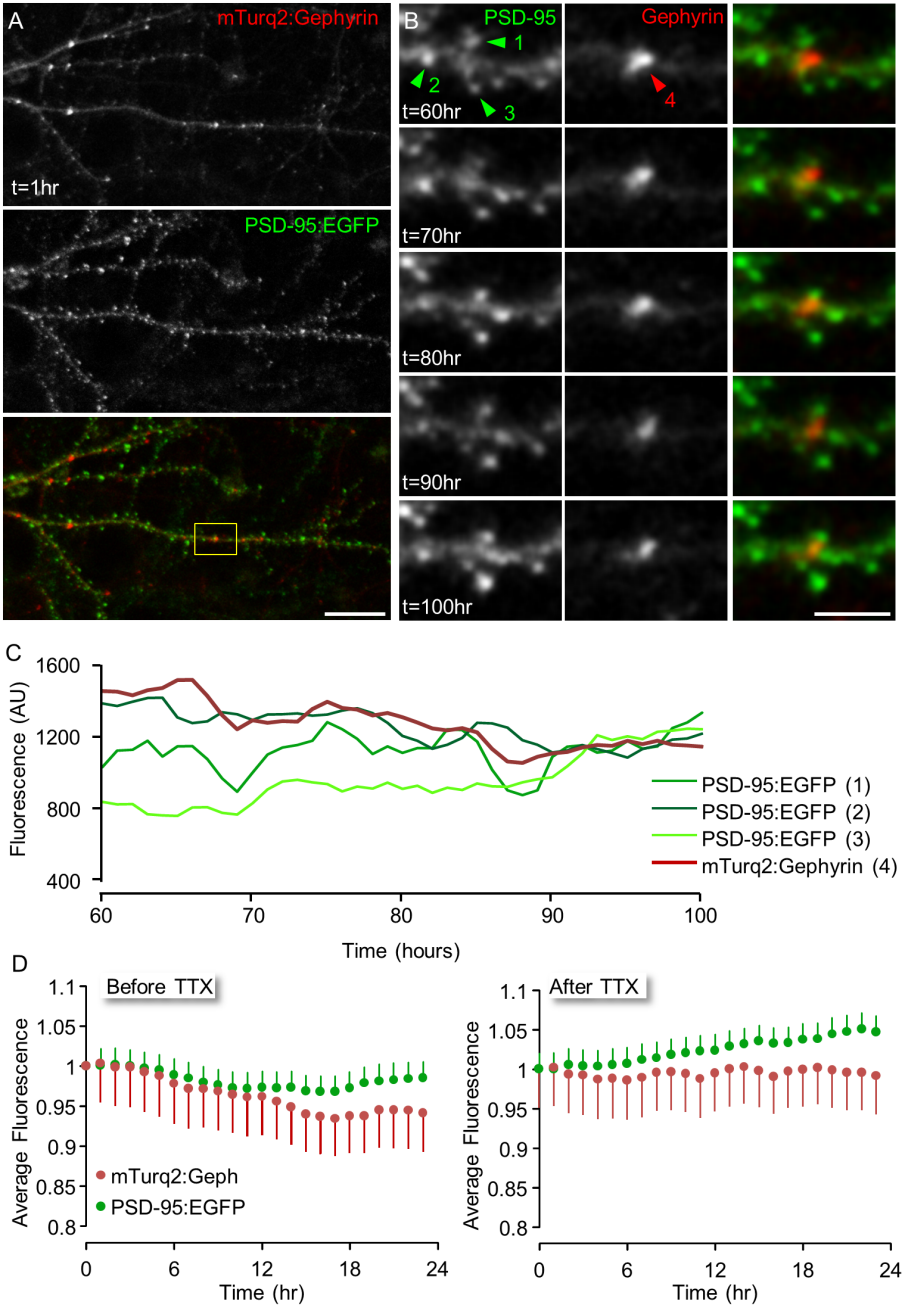
Concomitant imaging of GABAergic and glutamatergic synapses. **A)** A rat cortical neuron expressing both mTurq2:Geph and PSD-95:EGFP. **B)** Time-lapse imaging of the region enclosed in rectangle in (A). Images were collected at 1 hour intervals for 100 hours (only a subset of the data is shown here). Bars: 20μm (A), 5μm (B). **C)** Changes over time in the fluorescence intensity of one mTurq2:Geph and three neighboring PSD-95:EGFP puncta shown in (B) over 40 hours. **D)** Normalized fluorescence intensities of tracked PSD-95:EGFP and mTurq2:Geph puncta, 24 hours before (left) and after (right) exposure to TTX (4 Experiments, 6 neurons, 49 mTurq2:Gephyrin puncta and 163 PSD-95:GFP puncta, mean ± SEM).

To directly compare the spontaneous remodeling of GABAergic and glutamatergic synapses, we used lentiviral vectors to express, in the same neurons, two spectrally separable fusion proteins of postsynaptic scaffolding molecules, namely mTurq2:Geph for inhibitory synapses (as described so far) and PSD-95 fused to EGFP (PSD-95:EGFP) for glutamatergic synapses. PSD-95 is a core scaffold protein of glutamatergic synapses which has been used extensively to study the formation and remodeling of such synapses (e.g. [5,12,15,27,52–54]) and was shown to provide an excellent measure of excitatory postsynaptic density size [15,54]. One neuron in which both fusion proteins were expressed is shown in Fig 8A. As expected, the majority of PSD-95:EGFP puncta were located on dendritic spines, whereas inhibitory synapses were mainly located along dendritic shafts. The experiments were performed as described above and here too, TTX was introduced after 3–4 days of baseline recordings to examine the dependence on network activity. At the end of the experiments, nearby mTurq2:Geph and PSD-95:EGFP puncta were tracked separately and their fluorescence intensities obtained. To further minimize potential confounds that might arise from different locations along dendrites, we focused our attention on small groups of neighboring GABAergic and glutamatergic synapses as shown in Fig 8B for one mTurq2:Geph and three PSD-95:EGFP puncta.

We first examined whether the dual expression of both fluorescent reporters affected the gross dynamics observed for each of these when expressed alone. During 24 hour baseline recording periods, average fluorescence levels of mTurq2:Geph exhibited a modest decline whereas those of PSD-95:EGFP remained relatively stable. After TTX application, average mTurq2:Geph fluorescence intensities remained relatively stable, whereas those of PSD-95:EGFP gradually increased (Fig 8D). These observations are in good agreement with the data presented in Fig 4; (mTurq2:Geph) and prior studies ([5,41]; PSD-95:EGFP), indicating that the dual expression did not dramatically alter the gross dynamics of these fusion proteins.

**Fig 9:**
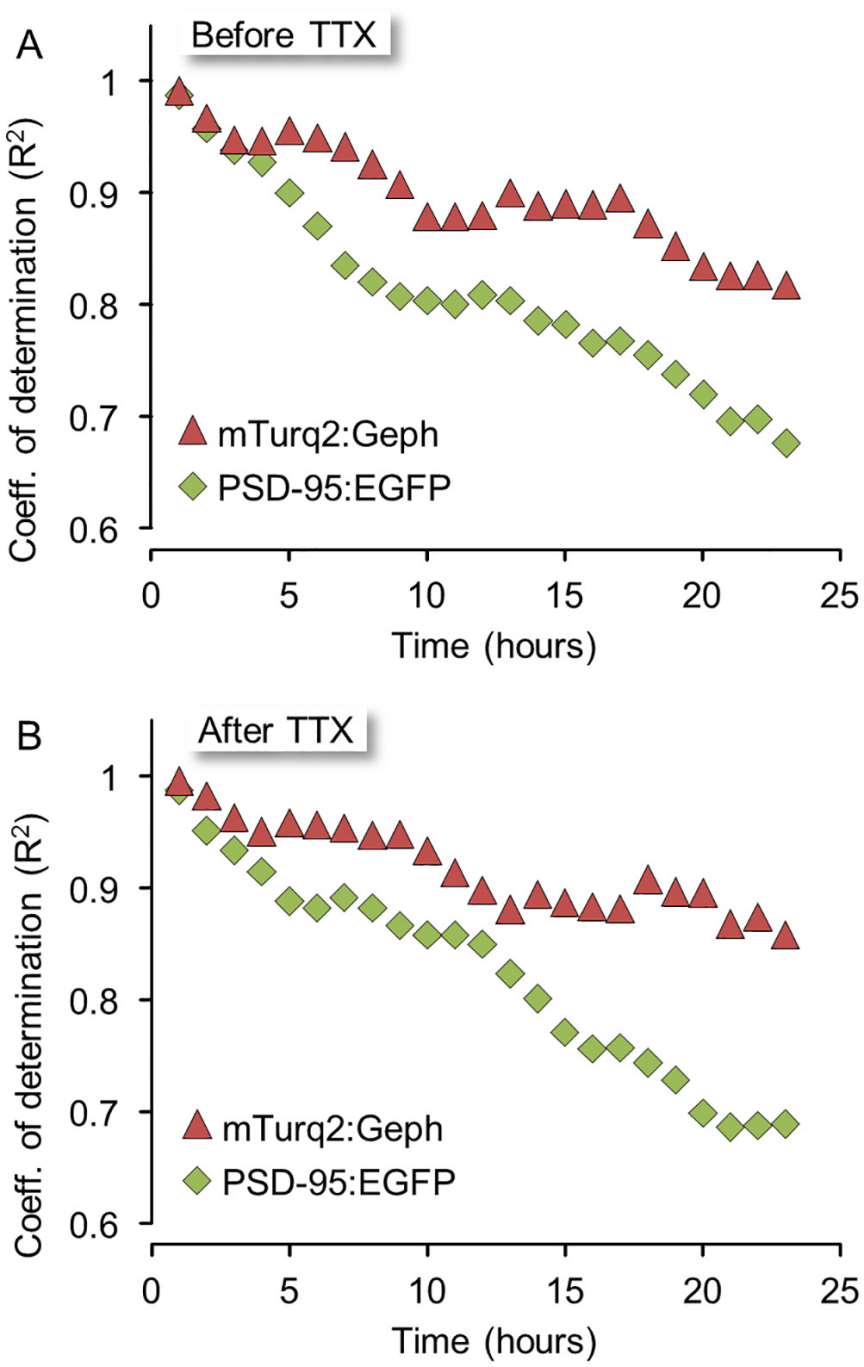
Inhibitory synapse configurations deteriorate more slowly than excitatory synapse configurations. **A)** Goodness of fit, expressed as the coefficient of determination (R^2^) of linear regression lines calculated as explained in Fig 6A. **B)** Same as in (A) but for 24 hour periods in TTX. Note the slower decay rates of slopes and R^2^ values for mTurq2:Geph in both active and silenced networks.

We then compared the deterioration rates of synaptic configurations for GABAergic and glutamatergic synapses positioned in the same neurons, at the same locations and thus, presumably, experiencing very similar histories. For each neuron expressing both reporters, 49 groups of inhibitory synapses and their neighboring excitatory synapses (in 6 neurons from 4 separate experiments) were selected and followed for 24 hours before and after TTX application as illustrated in Fig 8A–8C. We then plotted the fluorescence values of mTurq2:Geph and PSD-95:EGFP puncta, at increasing time intervals, as a function of their fluorescence at the beginning of each 24 hour window, in a manner similar to that shown in Fig 6A. The slopes in these plots decayed at faster rates for PSD-95:EGFP than they did for mTurq2:Geph, resulting in estimates of 〈*ε*〉 of 0.9916 and 0.9947 for PSD-95:EGFP and mTurq2:Geph, respectively (note that the latter is practically identical to the estimate of Fig 6). When this analysis was carried out on a cell to cell basis, however, differences between slope decay rates for PSD-95:EGFP and mTurq2:Geph did not reach statistical difference, possibly due to the difficulty of obtaining accurate estimates of 〈*ε*〉 from very small numbers of synapses. Importantly, however, goodness of fits (R^2^) values in these plots decayed much faster for PSD-95:EGFP in comparison to mTurq2:Geph, (Fig 9A and 9B) and this difference was statistically significant when carried out on a cell to cell basis (P = 0.045, paired t-test, n = 6 cells). As R^2^ is equal to the square of Pearson’s correlation coefficient, this difference suggests that inhibitory synaptic size configurations change at slower rates as compared to those of their excitatory counterparts.

**Fig 10:**
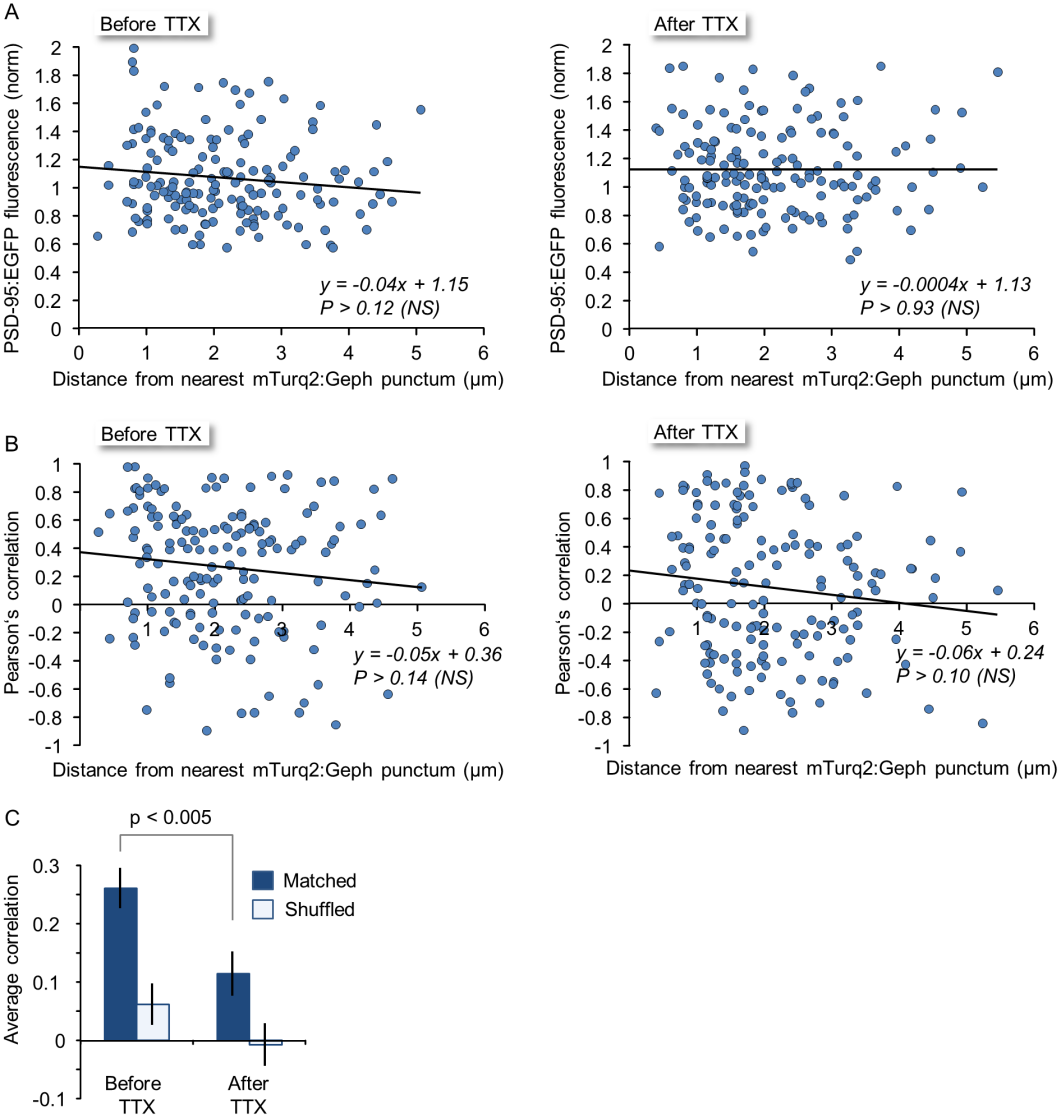
Relationships between the remodeling of nearby excitatory and inhibitory synapses. **A)** PSD-95:EGFP fluorescence intensity as a function of distance from nearest inhibitory synapse before (left) and after (right) exposure to TTX. NS = non-significant. **B)** Covariance (Pearsons’ correlation) of PSD-95:EGFP and mTurq2:Geph fluorescence as a function of distance between the synapses before (left) and after (right) exposure to TTX. **C)** Average (± SEM) covariance of PSD-95:EGFP and mTurq2:Geph within groups (but irrespective of distance) before and after exposure to TTX. Same synapses as in Fig 9. Open bars (“Shuffled”) correspond to the average (±SEM) covariance calculated for all possible pairs of PSD-95:EGFP and mTurq2:Geph in the entire data set (irrespective of neuron or experiment).

Several recent reports suggest that spontaneous remodeling of nearby inhibitory and excitatory synapses can be coupled (e.g. [36,27,55–57]). We wondered if this coupling, mainly observed at the level of synapse turnover, extends to spontaneous changes in synaptic sizes. We first examined whether a relationship could be found between PSD-95:EGFP fluorescence and the distance from the nearest inhibitory synapse. To that end, the fluorescence intensities of PSD-95:EGFP puncta were averaged over 24 hour windows, before and after TTX application. When these normalized intensities were plotted as a function of distance to the nearest mTurq2:Geph puncta (measured at the beginning of each time window), no discernible correlation was observed, neither before nor after TTX application (Fig 10A; 49 mTurq2:Geph puncta; 163 PSD95:EGFP puncta from 6 neurons in 4 experiments). We then examined to what degree changes in PSD-95:EGFP fluorescence co-varied with changes in mTurq2:Geph fluorescence in nearby inhibitory synapses and how this co-variance depended on the distance between them. To that end, we calculated the linear correlation (Pearsons’ correlation) between the fluorescence traces of nearby mTurq2:Geph and PSD-95:EGFP puncta over 24 hour windows and plotted these as a function of distance between the puncta (Fig 10B). Here too, no statistically significant correlation was observed, neither before not after TTX addition. However, when comparing the average co-variance within groups (regardless of distance) before and after TTX application, we did observe that the average correlation before TTX application was positive, and that it was significantly reduced following exposure to TTX (Fig 10C, Matched). As a control, we calculated the correlations of fluorescence traces between all mTurq2:Geph and PSD-95:EGFP puncta in the entire data set. As might be expected, no significant correlations were observed (Fig 10C, Shuffled). Thus, while some activity-dependent co-variance was observed for the remodeling of nearby excitatory and inhibitory synapse remodeling, no strong relationships with distance were observed, at least on the length scales examined here.

In summary, these data indicate that even though some weak, activity dependent coupling might exist between the spontaneous remodeling of nearby excitatory and inhibitory synapses, configurations of inhibitory synapses and excitatory synapses seem to deteriorate at different rates, with inhibitory synapses appearing to be less labile in this respect.

## Discussion

Here we set out to study the tenacity and spontaneous remodeling of inhibitory synapses and examine if the same principles previously shown to govern glutamatergic synapse remodeling dynamics also apply to inhibitory synapses. By following mTurq2:Geph expressed in cortical neurons for several days we found that the numbers and sizes of inhibitory synapses were generally stable as were the rightward skewed distributions of GABAergic synapse sizes. Blocking spontaneous network activity barely affected synapse numbers and sizes, had no lasting effect on either of these measures, and did not lead to the scaling of inhibitory synapse size distributions. At the same time, the sizes of inhibitory synapses within the same population changed significantly, with the extent of these remodeling dynamics unaffected by the complete suppression of spontaneous network activity. These spontaneous changes in synaptic sizes led to the gradual deterioration of inhibitory synapse configurations over time scales of several days in a manner that was consistent with a statistical framework previously developed to describe remodeling dynamics of glutamatergic synapses [14]. The rates at which inhibitory synapse configurations deteriorated did not seem to be affected by network activity as configurations deteriorated at similar rates even when network activity was completely suppressed. Finally, comparisons of remodeling dynamics for GABAergic and glutamatergic synapses in the same neurons and dendritic locations revealed that configurations of inhibitory synapses seem to be less labile than those of glutamatergic synapses, even though some degree of activity-dependent coupling was observed for the remodeling of nearby inhibitory and excitatory synapses. These findings thus point to certain differences in the spontaneous remodeling dynamics of GABAergic and glutamatergic synapses. Importantly, however, they also point to deep similarities in the processes that control synapse sizes and govern their spontaneous remodeling: The ongoing, apparently stochastic changes in individual synapse size, the resulting skewed shape and stability of synaptic size distributions, the gradual deterioration of synaptic configurations and the good fit to the same statistical process. The implications of these findings are described below.

### Experimental considerations

The assessment of inhibitory synapse size in this study was almost entirely based on quantifying the fluorescence of a fusion protein of gephyrin and mTurquoise2, with the assumption in mind that changes in mTurq2:Geph fluorescence reflect changes in GABAergic PSD size and, by extension, inhibitory synapse strength. This assumption has several caveats: First, unlike the excellent quantitative relationships established for glutamatergic synapses between the fluorescence of PSD-95 fusion proteins and electron microscopy-based measurements of PSD area (e.g. [15,54]), so far, to the best of our knowledge, similar relationships have not been established at this quantitative level of detail for gephyrin (but see [32]). Second, the very modest changes in synaptic gephyrin contents induced by prolonged activity suppression (in agreement with [58]) would seem to be at odds with other studies suggesting that activity-deprivation results in decreases in mIPSC amplitude and reductions in GABA_A_R labeling (e.g. [58–61]). We note, however, that in our hands no reductions in synaptic contents of the GABA receptor subunit γ2 were observed (see also [58,62]) and that others have reported opposite effects [44]. Finally, comparisons between mTurq2:Geph, GABA_A_ receptor subunits and presynaptic vesicular markers at individual synapses revealed that the correlations between these measures, while positive, were quite imperfect (Fig 2D and 2E). While good explanations for this imperfect correlation can be provided (see Results) it has to be acknowledged that relationships between gephyrin contents and inhibitory synapse strength are multilayered and not entirely obvious (see ref. [31] for example). Yet, given the critical role played by gephyrin in regulating the numbers of resident GABA_A_ receptors at GABAergic synapses, it is highly unlikely that synaptic gephyrin contents and synaptic strengths are unrelated ([39]; see also [25,34]). Indeed, a recent study showed that gephyrin recruitment is required for recruitment of GABA_A_ receptors during a form of inhibitory synapse long term potentiation [29] (see also [38]).

A more general concern relates to the experimental system used here, that is, networks of dissociated rat cortical neurons in primary culture. This system is advantageous in that it allows for excellent long-term optical and electrophysiological access, with the latter being extremely important to measure baseline network activity levels, verify the effects of pharmacological manipulations, and measure the “contrast” of activity features before and after such manipulations. Yet the question remains whether the limited tenacity of inhibitory synapses reported here is somehow related to our experimental system. It is worth noting, however, that large changes in the fluorescence of tagged gephyrin at inhibitory synapses have been recently reported *in vivo* as well [32]. Moreover, where excitatory synapses are concerned, spontaneous changes in synaptic contents of PSD-95:EGFP *in-vivo* ([15]; see also [63]) are at least as large as those observed in our *ex-vivo* networks ([5,14]; this study) indicating that our observations are not limited to cell culture settings, although particular rates measured in these two settings might differ.

### The tenacity of GABAergic synapses and its relationships with network activity

The experiments described above suggest that sizes of inhibitory synapses change spontaneously over time scales of hours and days. These observations are in excellent agreement with a recent study that examined spontaneous changes in presynaptic boutons of GABAergic neurons in organotypic hippocampal cultures [11]. In this study, it was shown that over time scales of hours, the volumes of individual presynaptic boutons exhibit very substantial fluctuations, comparable to those reported here for postsynaptic gephyrin. Similarly, despite these fluctuations, *average* bouton volumes remained generally stable over several hours. Furthermore, and in agreement with our own observations, manipulations of spontaneous activity levels had very modest effects on bouton volumes. Finally, and perhaps most striking are the observations on the transient effects of network activity suppression on bouton dynamics: whereas a 4 hour exposure to TTX suppressed bouton volume fluctuations, these returned to control levels after longer exposures, in excellent agreement with the transient effects we observed for postsynaptic gephyrin dynamics (Fig 7). These studies thus complement each other and indicate that the limited tenacity of GABAergic synapses is a synapse-wide phenomenon, in agreement with a prior report for excitatory synapses [12].

It might be asked how average synaptic sizes and synaptic size distributions remain constant when sizes of individual synapses change in an apparently stochastic fashion. As mentioned above, for populations of objects whose sizes fluctuate according to a Kesten process in the appropriate regime, size distributions are guaranteed to remain stable (see [14] for a more detailed explanation). Some intuition can be gained by examining Figs 6A, 7B, 7C, 7E and S5. As these figures show, even though synaptic sizes fluctuate in an apparently stochastic fashion, the combination of a negative (on average) scaling/multiplicative factor and a positive (on average) additive component leads to predictable tendencies: Larger than average synapses tend to grow smaller, whereas smaller than average synapses tend to grow larger. This powerful rule thus acts to maintain synaptic sizes within particular ranges even when synaptic sizes change in an apparently stochastic fashion [5,41]. It is also worth noting that the Kesten process also guarantees that such steady state size distributions will be rightward skewed, in concordance with the skewed size distributions observed here for inhibitory synapses (Fig 4) and those previously observed for glutamatergic synapses [5,7]. Interestingly, it has been suggested that the widespread occurrence of skewed distributions in brain organizational features has important implications for network function (reviewed in [64]); our finding concerning inhibitory size distributions would thus seem to further support this notion.

### Implications to network function

It is widely believed that the types, patterns, and strengths of synaptic connections formed among neurons determine the functional properties of neuronal networks or the information they contain. Moreover, changes in their properties or in their information content are generally believed to be realized by directed changes in patterns and strengths of synaptic connections. Increasing evidence, however, obtained both *in-vitro* and *in vivo* is leading to a gradual realization that excitatory synapses are not “static devices” that change only when instructed to do so, but also change spontaneously—even in the absence of particular activity patterns or any activity at all (reviewed in [2]). How such observations can be reconciled with prevailing notions on neuronal network function is not clear. An interesting result in this regard might be found in a recent study in which *in-vivo* measurements of spontaneous synaptic “volatility” were “plugged in” to simulations of large neuronal networks (Gianluigi Mongillo, Simon Rumpel, Yonatan Loewenstein, COSYNE 2015, abstract I-88). It was shown that key functional properties of such networks are much more sensitive to spontaneous changes in inhibitory connections as compared to spontaneous remodeling of excitatory ones, and it was concluded that inhibitory plasticity has a far larger potential for changing network functionality than the extensively-studied excitatory plasticity. Our findings that inhibitory synapse configurations are less labile than those of excitatory ones would seem to be in line with this idea, as they would imply that spontaneous changes in network function might be slower that what might be expected from observations of glutamatergic synapse “volatility”. Conversely, this finding might be interpreted to suggest that inhibitory synapses represent an effectively stable foundation that minimizes the impact of excitatory synapse size fluctuations, an interpretation that would be consistent with the relative insensitivity of inhibitory synapse remodeling to dramatic changes in network activity levels (see also [27]).

Although configurations of inhibitory synapses were less labile that those of their glutamatergic counterparts, such configurations changed nonetheless. Thus, quantitative differences notwithstanding, the emerging realization that excitatory synapse properties can change spontaneously seems to apply to inhibitory synapses as well. It is of particular note in this respect that major features of excitatory and inhibitory remodeling dynamics—the volatility of individual synapse sizes, the stability of their size distributions, the skewed shape of these distributions and the evolution of synaptic configurations—are all captured remarkably well by a statistical process that is essentially stochastic [14]. It thus remains to be understood how invariant function can be retained in networks composed of such highly unreliable components, a question that is not unrelated to one of the most fundamental issues in the life-sciences, that is the emergence of macroscopic order from microscopic disorder.

## Materials and Methods

### Neuronal cell cultures

Primary cultures of rat cortical neurons were prepared as described previously [5] using a protocol approved by the Technion committee for the supervision of animal experiments. Briefly, cortices of 1–2 days-old Wistar rats of either sex were dissected, dissociated by trypsin treatment followed by trituration using a siliconized Pasteur pipette. A total of 1–1.5x10^6^ cells were then plated on thin-glass multielectrode array (MEA) dishes (MultiChannelSystems—MCS, Germany) whose surface had been pre-treated with polyethylenimine (Sigma) to facilitate cell adherence. The preparations were then transferred to a humidified tissue culture incubator and maintained at 37°C in a gas mixture of 5% CO_2_, 95% air, and grown in medium containing minimal essential medium (MEM, Sigma), 25 mg/l insulin (Sigma), 20 mM glucose (Sigma), 2 mM L-glutamine (Sigma), 5 mg/ml gentamycin sulfate (Sigma) and 10% NuSerum (Becton Dickinson Labware). Half the volume of the culture medium was replaced three times a week with feeding medium similar to the medium described above but devoid of NuSerum, containing a lower L-glutamine concentration (0.5 mM) and 2% B-27 supplement (Invitrogen).

Primary cultures of rat hippocampal neurons for antibody labeling experiments were prepared as described previously [65]. Briefly, hippocampal CA1–CA3 regions of 1–2 days-old Wistar rats of either sex were dissected, dissociated as described above and plated onto 22×22 mm coverslips coated with poly-D-lysine (Sigma) inside 8-mm-diameter glass cylinder microwells (Bellco Glass). Culture medium consisted of MEM, 20 mM glucose, 0.1 g/l bovine transferrin (Calbiochem), 25 mg/l insulin (Sigma), 2 mM L-glutamine (Sigma), 10% NuSerum (Becton Dickinson Labware), 0.5% fetal bovine serum (HyClone), 2% B-27 supplement (Gibco), and 8 μM cytosine β-D-arabinofuranoside (Sigma) which was added to the culture medium after 3 days. Culture medium was replaced once a week with feeding medium.

### DNA constructs, lentivirus production and transduction

Exogenous DNA was introduced into neurons by means of a 3rd generation lentiviral expression system. These vectors code for the fluorescently tagged postsynaptic protein PSD-95:EGFP (described in [5]) and mTurq2:Gephyrin, constructed as described next.

Venus:Gephyrin in pEGFP-N1 [23] was provided as a generous gift by Antoine Triller (Ecole Normale Supérieure, Paris). The insert was sequenced and apart from one silent point mutation (Gly->Gly) and the absence of the N-terminal methionine was found to be identical to full length rat Gephyrin (NCBI Reference Sequence: NM_022865.3). A silent point mutation (His->His) was then made to eliminate an AgeI site, an XhoI site was added to 3' terminus of Gephyrin by PCR and the resulting construct was cut out and inserted between the BsrGI and XhoI sites of the FUGW lentiviral backbone [66] which we previously modified by moving the XhoI site from the 3’ to the 5’ side of the woodchuck hepatitis post-transcriptional regulatory element (WPRE). A construct coding for mTurquoise2 [40] flanked by AgeI and BsrGI was synthesized by large scale DNA synthesis. mTurquoise2 then was inserted into the modified FUGW backbone instead of EGFP between the AgeI and BsrGI sites. All cloning and gene synthesis was done by Genscript (Piscataway NJ, USA).

Lentiviral particles were produced by transfecting HEK293T cells with a mixture of three packaging plasmids: pLP1, pLP2, and pLP\VSVG (packaging vector mix of the ViraPower four-plasmid lentiviral expression system, Invitrogen) and the expression vector. HEK cell transfection was performed using Lipofectamine 2000 (Invitrogen) in 10cm plates when the cells had reached 80% confluence. Supernatant was collected after 48 hours, filtered through 0.45μm filters, aliquoted, and stored at -80°C.

Transduction of cortical cultures was performed at 5 days *in-vitro* (DIV) by adding predetermined amounts of the filtered supernatant to each MEA dish.

### Electrophysiological recordings

A MEA system was used to continuously monitor the electrical activity of the network through 59, 30 μm diameter recording electrodes, arranged in an 8x8 array, spaced 200 μm apart. The dishes contain 59 rather than 64 electrodes because the corner electrodes are missing, and one of the remaining leads is connected to a large substrate embedded electrode designed to be used as a reference (ground) electrode. The flat, round electrodes are made of titanium nitride, whereas the tracks and contact pads are made of transparent Indium Tin Oxide.

Network activity was recorded through a commercial 60-channel headstage/amplifier (Inverted MEA1060, MCS) with a gain of 1024x. The amplified signal was multiplexed into 16 channels, amplified by a factor of 10 by a 16 channel amplifier (Alligator technologies, USA) and then digitized by an A/D board (Microstar Laboratories, USA) at 12 KSamples/sec per channel. Data acquisition was performed using AlphaMap (Alpha-Omega, Israel). All data was stored as threshold crossing events with the threshold set to -40μV. Electrophysiological data were imported to Matlab (MathWorks, USA) and analyzed using custom written scripts.

### Long-term imaging

All imaging was performed on neurons grown on thin glass MEA dishes as described above. These particular MEA dishes are fabricated of very thin glass (180 μm), which allows for the use of high numerical aperture, oil immersion objectives and are thus ideally suited for high-resolution imaging [5]. Scanning fluorescence and brightfield images were acquired using a custom designed confocal laser scanning microscope based on a Zeiss Axio Observer Z1 using a 40×, 1.3 N.A. Fluar objective. The system was controlled by custom written software and includes provisions for automated, multisite time-lapse microscopy. MEA dishes containing networks of cortical neurons were mounted on the aforementioned headstage/amplifier which was attached to the microscope’s motorized stage.

mTurq2:Gephryin and PSD-95:EGFP were excited using 457 nm (Cobolt) and 488 nm (Coherent) solid state lasers, respectively. Fluorescence emissions were read through 467–493 nm and 500–550 nm bandpass filter (Semrock, USA and Chroma Technology, USA). Time-lapse recordings were usually performed by averaging six frames collected at each of 8 focal planes spaced 0.8 μm apart. All data were collected at a resolution of 640 x 480 pixels, at 12 bits/pixel, with the confocal aperture fully open. Data was collected sequentially from up to 12 predefined sites, using the confocal microscope robotic XYZ stage to cycle automatically through these sites at 1 hour (or 24 hour) time intervals. Focal drift during the experiment was corrected automatically by using the microscopes' "autofocus" feature.

### Experimental conditions in long term experiments

MEA dishes were covered with a custom designed cap containing inlet and outlet ports for perfusion media and air mixtures, a reference ground electrode and a removable transparent glass window. The MEA dish was continuously perfused with feeding media (described above) at a rate of 2.5 ml/day by means of a custom built perfusion system based on an ultra-slow flow peristaltic pump (Instech Laboratories Inc., USA) and silicone tubing. The tubes were connected to the dish through the appropriate ports in the custom designed cap. A 95% air / 5% CO_2_ sterile mixture was continuously streamed into the dish at very low rates through a third port with flow rates regulated by a high precision flow meter (Gilmont Instruments, USA). The base of the headstage/amplifier and the objective were heated to 37°C and 36°C respectively using resistive elements, separate temperature sensors and controllers, resulting in temperatures of 36–37°C in the culture media.

### Imaging data analysis

All imaging data analysis was performed using custom written software (“OpenView”). Special features of this software allow for automated / manual tracking of individual synaptic puncta and measurements of fluorescent intensities of these over time (described in detail in [41]). 9 × 9 pixel (~1.3 x 1.3μm) areas were placed on the centers of fluorescent puncta and mean pixel intensities within these areas were obtained from maximal intensity projections of Z section stacks.

For measuring distributions of puncta intensities, areas were placed programmatically on fluorescent puncta at each time step using identical parameters but no tracking of individual puncta was performed (see Fig 3C, bottom panel). For tracking identified puncta, areas were placed initially over all puncta and then a smaller subset (typically 30–50 per site) was thereafter tracked. As the reliability of automatic tracking was not absolutely perfect, all tracking was verified and, whenever necessary, corrected manually. Puncta for which tracking was ambiguous were excluded.

Because mTurq2:Geph (and PSD-95:EGFP) expression levels varied slightly from one neuron to another, mTurq2:Geph (and PSD-95:EGFP) puncta fluorescence data from each neuron were first normalized to mean mTurq2:Geph (or PSD-95:EGFP) puncta fluorescence of that neuron, allowing us to pool data from different neurons and experiments, correcting for differences in expression levels and potential variations in optical parameters between experiments. Consequently, all fluorescence values throughout the study are expressed as fractions of mean neuronal mTurq2:Geph (or PSD-95:EGFP) fluorescence at initial time points.

### Pharmacological manipulations

Tetrodotoxin (TTX; Alomone Labs) was diluted in 100 μl of medium drawn from the culture dish while on the microscope. The mixture was subsequently returned to the dish and mixed gently. Applications to the dish were complemented by simultaneous addition to the perfusion media. Final concentrations in the dish and perfusion media were 1 μM. In one experiment, bicuculline (Sigma) was added as well.

### Live cell antibody labeling

Primary antibodies against extracellular epitopes of the GABA_A_R γ2 or β2,3 subunits (rabbit: 1:500; Synaptic Systems, Gottingen, Germany; mouse: 1:100; Millipore) were prepared in phosphate buffered saline (PBS) at the predetermined concentration. The primary antibodies were labeled with fluorescent Fab’ fragments (Zenon labeling kit; Invitrogen) according to the manufactures instructions (Zenon 647nm—rabbit and Zenon 488—mouse, respectively). At 14–18 DIV, primary hippocampal cultures were incubated for 1 hour at 37°C with the primary/Fab’ mixtures (antibodies against GABA_A_R γ2 or β2,3 subunits) or a primary antibody against Vesicular GABA transporter (VGAT) lumenal domain fluorescence-labeled with Oyster 488 (rabbit: 1:200; Synaptic Systems [42]). After the incubation period, the cells were gently washed three times in physiological solution ("Tyrode's", 119mM NaCl, 2.5mM KCl, 2mM CaCl_2_, 25mM HEPES, 30mM glucose, buffered to pH 7.4) and imaged immediately.

## Supporting Information

S1 Fig Spontaneous activity in networks of cortical networks. **A)** A two minute raster plot of action potentials recorded from the MEA 59 electrodes. Each dot represents a single action potential. Recording is from the same experiment shown in Fig 4A (~72 hours from beginning of experiment). **B)** An enlarged, 2-second portion of the trace shown in A.
(TIF)

S2 Fig Changes in population measures of inhibitory synapses in a single experiment. **A)** Changes in the number of mTurq2:Geph puncta in 6 neurons in the same experiment as in Fig 4A over the same 72 hour period. Counts for each neuron were normalized to initial puncta counts at t = 0. **B)** Mean fluorescence intensities of mTurq2:Geph puncta in the same 6 neurons as in A) (~266 synapses). Fluorescence values were normalized to mean fluorescence at t = 0. **C)** The slight decline in mTurq2:Geph puncta fluorescence is not due to photobleaching. Changes in mTurq2:Geph puncta fluorescence intensities pooled from 16 neurons in 2 experiments in which images were obtained at 24 hour instead of one hours intervals. All values in represent means ± standard deviations.
(TIF)

S3 Fig Spontaneous activity 46 hours before and after the addition of TTX (1 μM) to the MEA dish and perfusion medium. Activity was normalized to baseline activity levels at t = -24 hours. Average of 4 experiments.
(TIF)

S4 Fig The effects of activity suppression on population measures of inhibitory synapses are not altered by blocking GABA_A_ receptors with bicuculline. **A)** Changes in the number of mTurq2:Geph puncta in a single experiment in which bicuculline (6 μM) was added together with TTX. Counts for each neuron were normalized to initial puncta counts at t = 0. ~210 synapses from 8 neurons. **B)** Mean fluorescence intensities of mTurq2:Geph puncta for the same 8 neurons as in A). Note the similarity with Fig 4C and 4D.
(TIF)

S5 Fig The fluorescence of individual mTurq2:Geph puncta as a function of their fluorescence 12 hours beforehand. **A)** Before exposure to TTX. **B-D)** After exposure to TTX. Solid lines are linear regression fits. Unity lines are shown as dashed red lines. Note the transient change in regression line trends during the 12 hour window following exposure to TTX (B), and the recovery of these trends in subsequent time windows (C,D). Same data as in Fig 7.
(TIF)

S1 Dataset A Microsoft Excel spreadsheet containing raw data used to generate the major figures. The figure to which data pertains to is indicated in the tab of each worksheet. Data are provided as fractions of mean fluorescence (i.e. as a fraction of the mean fluorescence measured for the particular neuron each synapse belongs to). All times are in hours. For the data of Figs 5–10, data are after smoothing with a 3 point low pass filter.
(XLSX)
