# Supplementary figures and images for: Remodeling and Tenacity of Inhibitory Synapses: Relationships with Network Activity and Neighboring Excitatory Synapses
Source: PLoS Comput Biol. 2015 Nov 24;11(11):e1004632. doi: 10.1371/journal.pcbi.1004632 (PMC4658206; doi:10.1371/journal.pcbi.1004632)

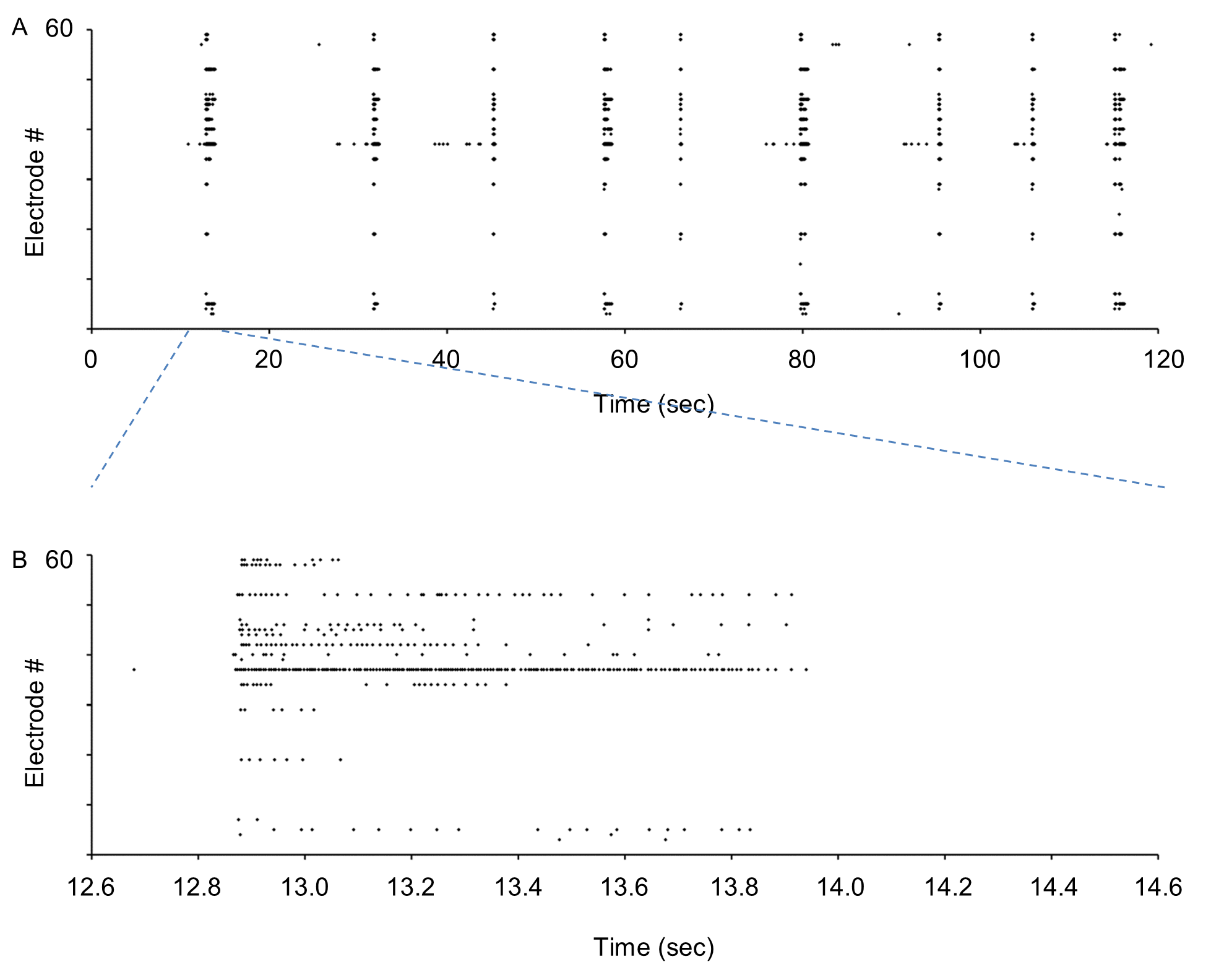

Supplement: S1 Fig — A) A two minute raster plot of action potentials recorded from the MEA 59 electrodes. Each dot represents a single action potential. Recording is from the same experiment shown in Fig 4A (~72 hours from beginning of experiment). B) An enlarged, 2-second portion of the trace shown in A. (TIF) [file pcbi.1004632.s001.tif]

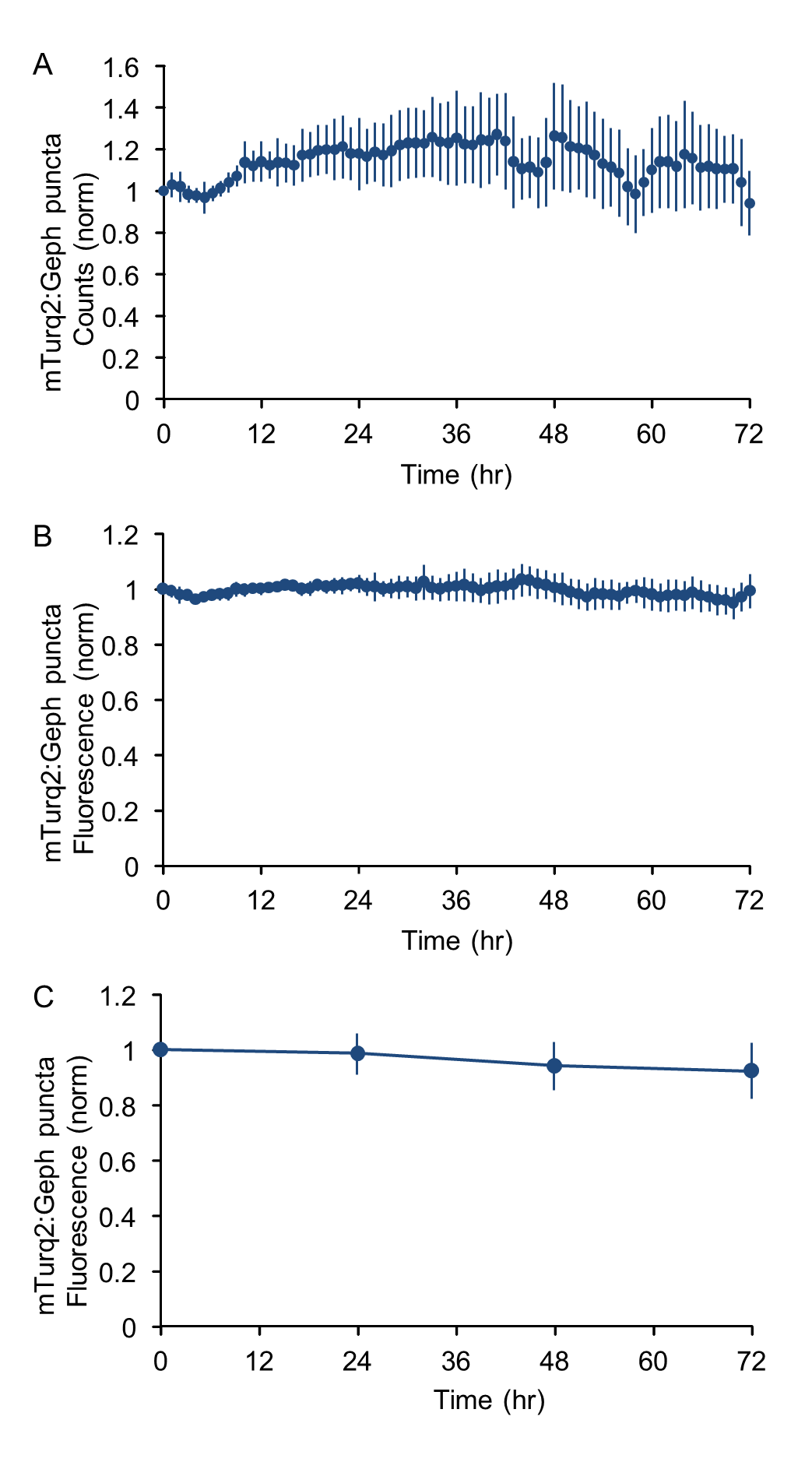

Supplement: S2 Fig — A) Changes in the number of mTurq2:Geph puncta in 6 neurons in the same experiment as in Fig 4A over the same 72 hour period. Counts for each neuron were normalized to initial puncta counts at t = 0. B) Mean fluorescence intensities of mTurq2:Geph puncta in the same 6 neurons as in A) (~266 synapses). Fluorescence values were normalized to mean fluorescence at t = 0. C) The slight decline in mTurq2:Geph puncta fluorescence is not due to photobleaching. Changes in mTurq2:Geph puncta fluorescence intensities pooled from 16 neurons in 2 experiments in which images were obtained at 24 hour instead of one hours intervals. All values in represent means ± standard deviations. (TIF) [file pcbi.1004632.s002.tif]

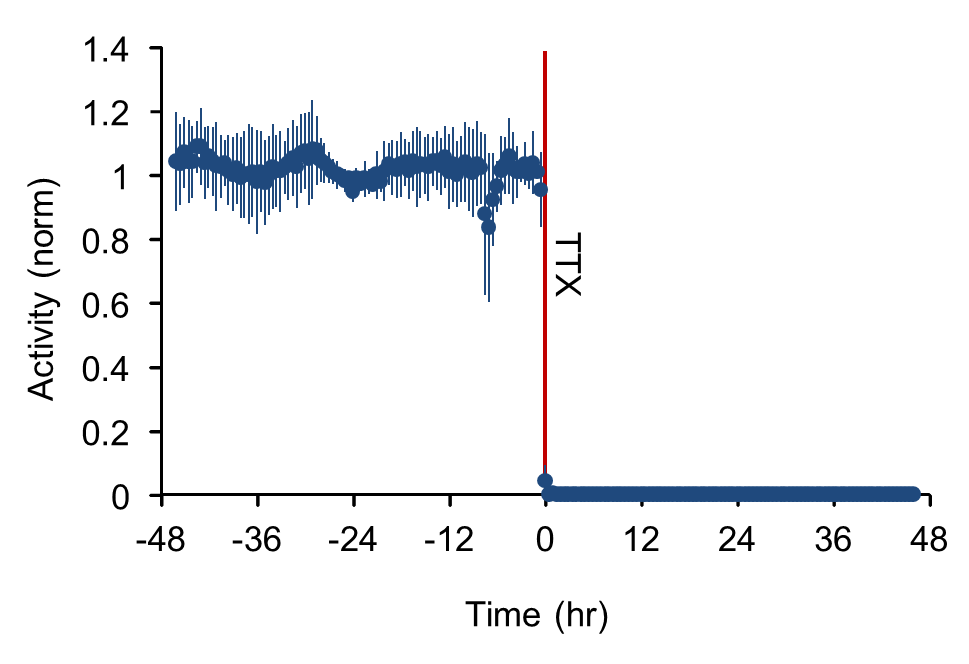

Supplement: S3 Fig — Activity was normalized to baseline activity levels at t = -24 hours. Average of 4 experiments. (TIF) [file pcbi.1004632.s003.tif]

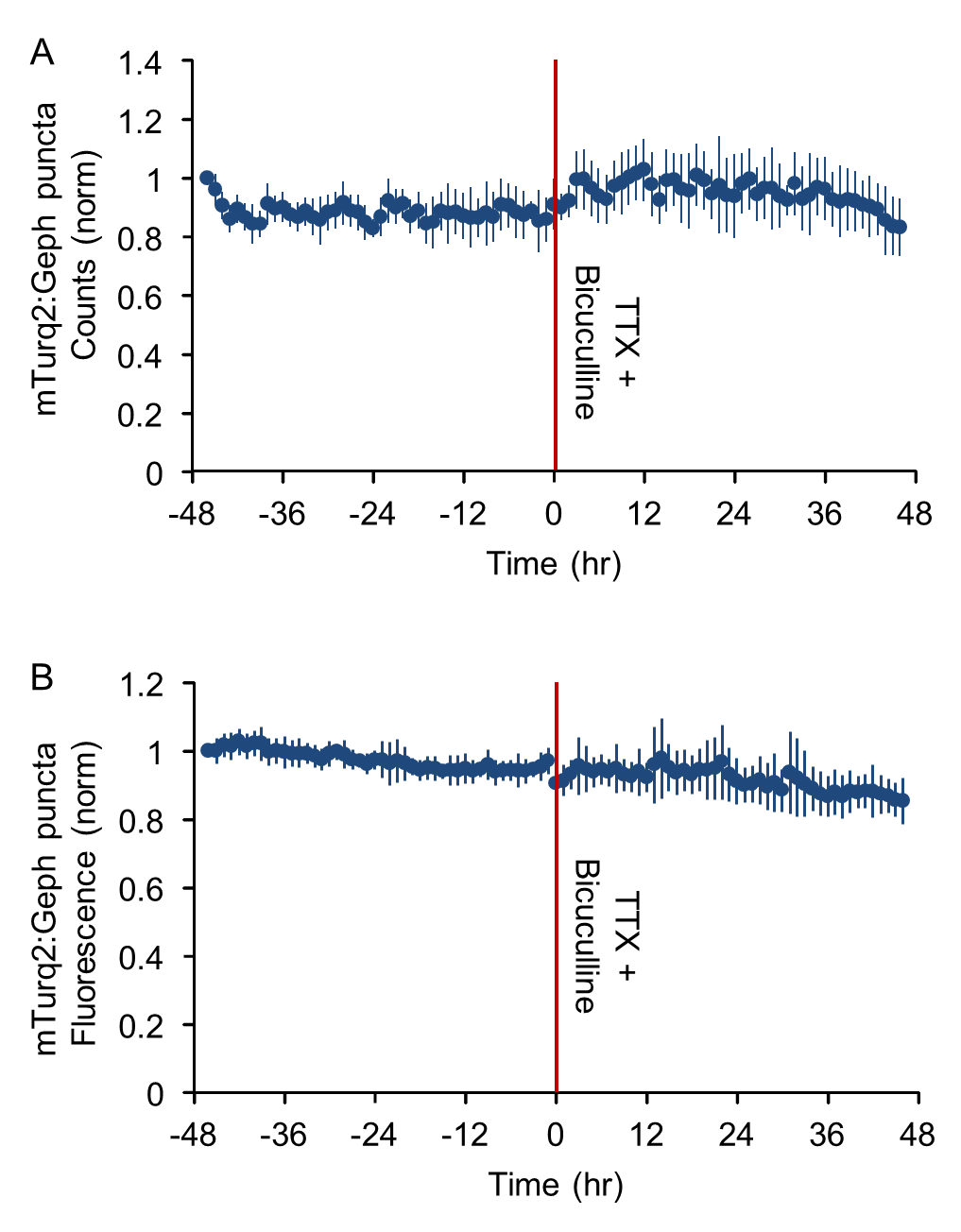

Supplement: S4 Fig — A) Changes in the number of mTurq2:Geph puncta in a single experiment in which bicuculline (6 μM) was added together with TTX. Counts for each neuron were normalized to initial puncta counts at t = 0. ~210 synapses from 8 neurons. B) Mean fluorescence intensities of mTurq2:Geph puncta for the same 8 neurons as in A). Note the similarity with Fig 4C and 4D. (TIF) [file pcbi.1004632.s004.tif]

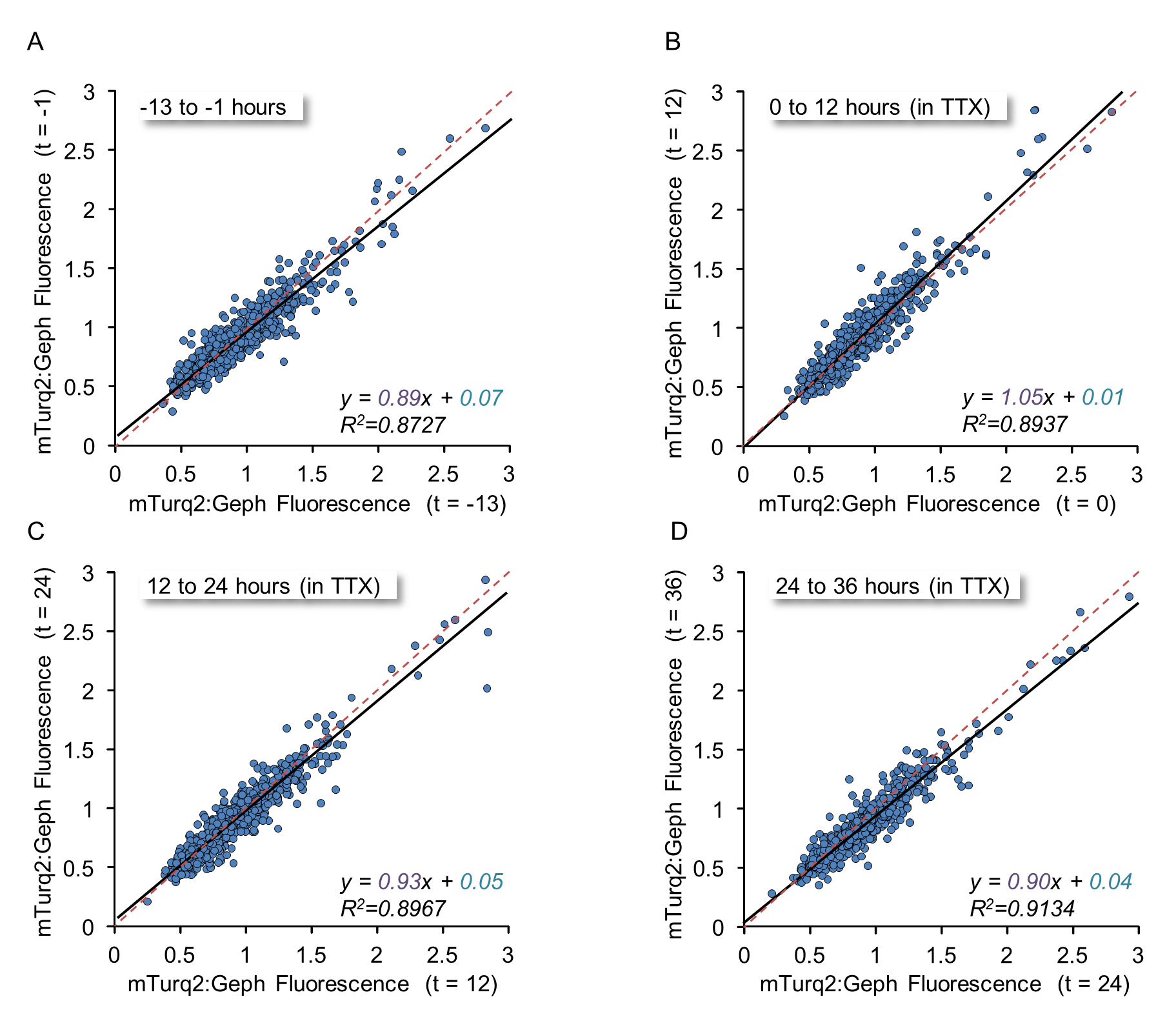

Supplement: S5 Fig — A) Before exposure to TTX. B-D) After exposure to TTX. Solid lines are linear regression fits. Unity lines are shown as dashed red lines. Note the transient change in regression line trends during the 12 hour window following exposure to TTX (B), and the recovery of these trends in subsequent time windows (C,D). Same data as in Fig 7. (TIF) [file pcbi.1004632.s005.tif]
